# Limitations of biopsy-based transcript diagnostics to detect T-cell-mediated allograft rejection

**DOI:** 10.1093/ndt/gfae147

**Published:** 2024-06-26

**Authors:** Lukas Weidmann, Dusan Harmacek, Kai Castrezana Lopez, Birgit Maria Helmchen, Ariana Gaspert, Raphael Korach, Nicola Bortel, Nicolas Schmid, Seraina von Moos, Elena Rho, Thomas Schachtner

**Affiliations:** University Hospital Zurich, Department of Nephrology, Zurich, Switzerland; University Hospital Zurich, Department of Nephrology, Zurich, Switzerland; University Hospital Zurich, Department of Nephrology, Zurich, Switzerland; University Hospital Zurich, Department of Pathology and Molecular Pathology, Zurich, Switzerland; University Hospital Zurich, Department of Pathology and Molecular Pathology, Zurich, Switzerland; University Hospital Zurich, Department of Nephrology, Zurich, Switzerland; University Hospital Zurich, Department of Nephrology, Zurich, Switzerland; University Hospital Zurich, Department of Nephrology, Zurich, Switzerland; University Hospital Zurich, Department of Nephrology, Zurich, Switzerland; University Hospital Zurich, Department of Nephrology, Zurich, Switzerland; University Hospital Zurich, Department of Nephrology, Zurich, Switzerland

**Keywords:** Banff classification, borderline changes, isolated arteritis, MMDx, TCMR-suspicion

## Abstract

**Background:**

Isolated tubulitis, borderline changes and isolated arteritis suspicious for histologic T-cell-mediated rejection (hTCMR) remain findings of uncertain significance. Although the Molecular Microscope Diagnostics System (MMDx) has not been trained on those lesions, it was suggested that MMDx might reclassify a subgroup to molecular TCMR (mTCMR).

**Methods:**

In this single-center cohort of 326 consecutive, unselected kidney allograft biopsies assessed by histology and MMDx, we analyzed 249 cases with isolated tubulitis (i0, t1–3, v0; *n* = 101), borderline changes (according to Banff 2022, v0; *n* = 9), isolated arteritis (no borderline, v1; *n* = 37), no inflammation (i0, t0, v0; *n* = 67) and a positive control cohort (hTCMR, *n* = 27; mixed histologic rejection, *n* = 8; both according to Banff 2022; total *n* = 35). The first three groups were summarized as TCMR-suspicion (*n* = 147). Subcategorization included the presence and absence of microvascular inflammation (MVI); g+ptc ptc ≥2. Molecular rejection rates and differentiation were investigated.

**Results:**

Molecular rejection rates were 37/147 cases (25.2%; 32 with MVI) in TCMR-suspicion, 6/67 (9%; 4 with MVI) in no inflammation and 30/35 (85.7%; 19 with MVI) in the positive control cohort. Molecular antibody-mediated rejection (mAMR) was present in 39/73 (53.4%) of cases. The presence of donor-specific antibodies at the time of the biopsy was high (127/249, 51%). Only 3 mAMR/TCMR and 0 pure mTCMR cases were detected in TCMR-suspicion and no inflammation, compared with 12 mAMR/TCMR and 10 mTCMR cases in the positive control cohort (*P* < .001). Even though the TCMR-specific molecular (Classifier) score differentiated between TCMR-suspicion and no inflammation (*P* = 0.005), rejection phenotype scores (R2 and R3) did not (*P* = .157 and .121).

**Conclusions:**

MMDx did not identify pure mTCMR among isolated tubulitis, borderline changes or isolated arteritis, likely due to low sensitivity for TCMR lesions. However, it identified mAMR or mAMR/TCMR, especially in cases with MVI. Subthreshold findings remain to be further studied.

## Graphical Abstract

**Graphical Abstract:**
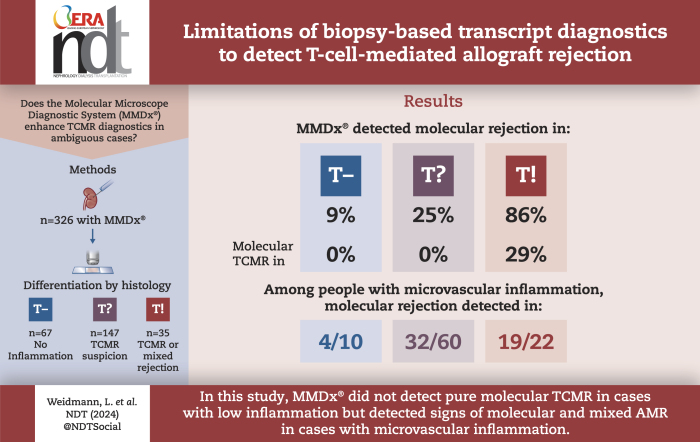

KEY LEARNING POINTS **What was known:**

- The Molecular Microscope Diagnostic System (MMDx) is an established biomarker in kidney allograft rejection diagnosis, particularly in the setting of antibody-mediated rejection (AMR), and has been integrated into the Banff classification.
- Prior studies about MMDx brought attention to limitations, such as discrepancies with histologic evaluations and uncertainty in early-stage alloimmune injury and overlapping pathologies.
- The clinical utility of MMDx in cases where histological findings raise suspicion for histologic T-cell-mediated rejection (hTCMR) remains understudied.

**This study adds:**

- A comprehensive investigation of the practical application of MMDx within a large kidney transplant biopsy cohort at a European transplant center.
- Focus on exploring MMDx in cases where TCMR was suspected based on histologic evidence but did not align with all Banff criteria for hTCMR diagnosis, with a diligent process of excluding any overlapping pathologies beforehand

**Potential impact:**

- This study highlights the low added value of MMDx in identifying mTCMR in cases when histologic suspicion is present, which may have implications for future diagnostic guidelines.
- It emphasizes the potential utility in diagnosing concomitant mAMR/TCMR or mAMR.
- Lastly, our study aims to provide clinicians with guidance on when an MMDx analysis may be of assistance.

## INTRODUCTION

Kidney transplant biopsies remain the cornerstone to diagnosing acute allograft rejection in kidney transplant recipients (KTRs) and are performed when alloimmune injury is suspected [1–3]. Although the introduction of the Banff classification as a guideline for histological rejection diagnosis offered more objective criteria based on defined lesions (i-, t-, v-, g-, ptc-, etc.), the semiquantitative assessment holds the problem of interobserver variability [4–7]. T-cell-mediated rejection (TCMR), as one modality of rejection, is diagnosed based on three histological Banff lesions: interstitial inflammation (i-lesion), tubulitis (t-lesion) and intimal arteritis (v-lesion) [8]. Decisions for management vary according to the severity of TCMR: treatment protocols for fully developed TCMR include pulsed steroids and/or anti-thymocyte globulin (ATG), as well as an optimization of maintenance immunosuppression. These protocols were established many years ago [9, 10]. However, management in cases of borderline changes or isolated TCMR lesions remains unclear. Additionally, several important lesions of the TCMR continuum (i-, t-, v-lesions) have confounding factors, especially if the occurrence is standalone: i- and t-lesions are found in acute kidney injury (AKI) and other overlapping pathologies (BK nephropathy, pyelonephritis, acute interstitial nephritis) [11, 12]. Intimal arteritis (v-) lesions are not specific for TCMR or antibody-mediated rejection (AMR), and their relevance for clinical outcomes as isolated lesions remain equivocal [13, 14]. In fact, isolated v-lesions in the early phase after transplantation could be representative of ischemic changes rather than acute vascular rejection [15]. Different biomarkers other than the Banff classification, such as the donor-derived cell-free DNA (DD-cfDNA), the Banff Human Organ Transplant Panel (B-HOT) or quantitative polymerase chain reaction (qPCR) are currently in discussion and used for the exclusion or inclusion of rejection, and have been proven to be useful, especially in reclassifying biopsies suspicious for AMR [16–21]. The development and validation of the Molecular Microscope Diagnostic System (MMDx) as a microarray-based mRNA assessment, also offered value in the diagnosis of TCMR and AMR [22–24]. However, although independent of individual bias by the pathologist, limitations like discrepancies with histology and uncertainty of MMDx in early stages of alloimmune injury or overlapping pathologies have been addressed in previous research, and its validation in clinical practice in such cases is still needed [25]. A recent study showed no identification of mAMR in cases with donor-specific antibodies (DSAs) but without microvascular inflammation (MVI) in an independent cohort from the validation cohort [26]. Earlier studies also showed variable results of molecular diagnosis in cases of histological uncertainty regarding TCMR-suspicion—yet it was suggested that MMDx may be able to reclassify molecular TCMR in a subset of patient groups with either/or i-, t- and v-lesions that do not meet all Banff criteria for diagnosis of TCMR [27–31]. In our cohort from the University Hospital of Zurich we investigated the added value of MMDx in ambiguous histologic lesions of the TCMR continuum, i.e. isolated tubulitis (i0, t1–3, v0), borderline changes (according to Banff 2022) and isolated arteritis lesions (no borderline, v1) with prior exclusion of influencing factors, such as overlapping pathologies, pre-treatment or concurrent chronic-active TCMR (caTCMR).

## MATERIALS AND METHODS

### Study population

This single-center study consists of 249 kidney transplant biopsies (21 protocol, 228 for cause) from 219 KTRs, consecutively and unselectively conducted between 2021 and 2023 at the University Hospital of Zurich. All patients gave general consent for using clinical data, including biopsy results and MMDx analysis. The study was approved by the cantonal ethics commission review board of Zurich, Switzerland (BASEC 2020-02 817) and complied with the Declaration of Helsinki.

### Rejection terminology

In the context of explaining rejections based on histology throughout the manuscript, the prefix ‘h’ is appended (e.g. hAMR). However, when referring to molecular phenotypes of rejections, the prefix ‘m’ is used (e.g. mAMR).

### Biopsy process and histologic categorization

**Figure 1:**
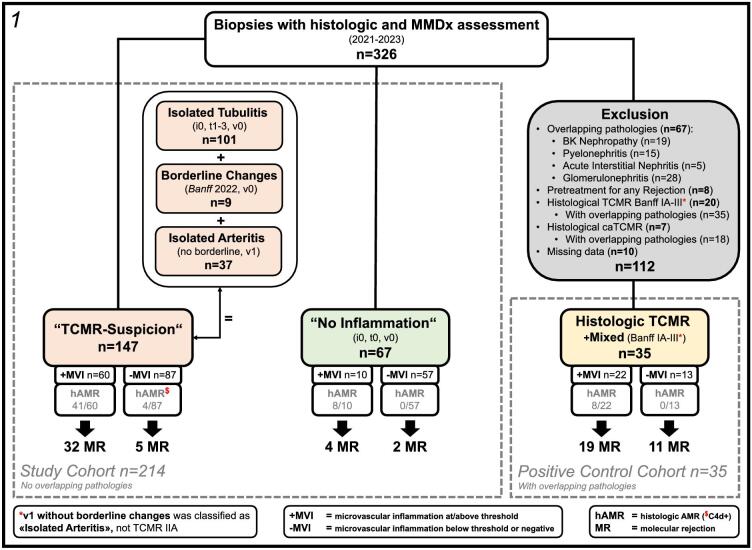
Deduction and categorization of the whole cohort: 249 biopsies were drawn out of 326 biopsies after the exclusion of competitive findings. Categorization was performed into a TCMR-suspicion group (*n* = 147) containing cases with isolated tubulitis (i0, t1–3, v0; *n* = 101), borderline changes (according to Banff 2022, v0; *n* = 9) and isolated arteritis (no borderline, v1; *n* = 37). Furthermore, a group with no inflammation (i0, t0, v0; *n* = 67) and a positive control cohort (hTCMR and mixed histologic rejection; *n* = 35) were deducted from the primarily excluded biopsies. Overall, 73 cases of molecular rejections were observed (37 from within TCMR-suspicion, 6 from within no inflammation and 30 from within the positive control cohort).

All biopsies were evaluated at the bedside for adequacy (sufficient cortex) during the procedure. Local pathologists assigned histologic diagnoses according to the 2018 Reference Guide to the Banff Classification and The Banff 2019 Kidney Meeting Report [5, 8]. Our study team reclassified all biopsies according to the Banff update 2022 [32]. The study cohort (*n* = 214) was categorized into isolated tubulitis (*n* = 101), borderline changes (*n* = 9), isolated arteritis (*n* = 37) and a group without suspicion for TCMR (i0, t0, v0; no inflammation, *n* = 67), the latter most importantly for comparison of molecular (Classifier) and rejection phenotype scores (R scores). Thirty-five cases were included as a positive control cohort consisting of either hTCMR (divided into TCMR IA/IB, *n* = 9 or TCMR IIA/IIB, *n* = 18; total *n* = 27) or mixed histologic rejection (hAMR/TCMR; *n* = 8). Inclusion criteria for all 249 biopsies were: biopsies with a complete histologic examination based on relevant Banff lesions (i-, t-, v-, g-, ptc-lesions) according to the Banff classification and corresponding MMDx analysis. Exclusion criteria for the study cohort (*n* = 214) were hTCMR (including caTCMR) or hAMR/TCMR according to the Banff classification, and alternative histopathologic findings competing with T-cell-specific Banff lesions or glomerulitis (BK nephropathy, pyelonephritis, acute interstitial nephritis and glomerulonephritis; *n* = 67). Patients receiving anti-rejection therapy shortly before biopsy were also excluded (*n* = 8). Isolated v1 (formally TCMR IIA regardless of i- and t-lesions) was an exception, as it was classified as isolated arteritis. As the main subcategorization of all subgroups was presence or absence of microvascular inflammation (MVI), hAMR cases were also included. The detailed deduction is demonstrated in Fig. 1.

### Definitions according to the Banff classification

Isolated tubulitis was defined as no foci of inflammation (i0) or arteritis (v0) but foci of tubulitis (t1–3). Borderline changes followed the diagnostic criteria of the Banff update 2022 [8]. Importantly, while a formal classification would designate the v1-lesion as TCMR IIA regardless of i- and t-lesions, it was categorized as isolated arteritis in this study, if no criteria for borderline changes (i > 0 and t > 0) were present. hTCMR criteria in the positive control cohort also followed the diagnostic criteria of Banff 2022. MVI at or above threshold was defined as g+ptc ≥2, with g >0; ptc2 only in cases without borderline changes or hTCMR (*n* = 3). g+ptc >0, but <2 was considered MVI below threshold and g+ptc = 0 negative. C4d was counted as negative in ABO-incompatible transplantations (total *n* = 19) and counted as positive if >1 (tested by immune fluorescence). Further subcategorization based on the new AMR continuum presented in the Banff update 2022 consisted of: findings below threshold, probable hAMR (DSA+, C4d–, g+ptc <2 but >0), DSA-negative, C4d-negative, MVI (g+ptc ≥2) and hAMR [32].

### MMDx analysis

Small tissue samples (3–4 mm) were obtained from one of the at least two biopsy cores and stored in RNAlater^®^ solution. MMDx analysis was performed in Kashi Clinical Laboratories (Portland, OR, USA) per protocol. The following molecular rejection phenotypes were assessed: mTCMR, mAMR/TCMR, minor mAMR (rejection score “normal,” but AMR score “mild/moderate”) and mAMR. Rejection score “mild/moderate,” but AMR and TCMR scores “normal” (*n* = 6), and rejection score “normal,” but TCMR score “mild” (*n* = 1) were counted as “no molecular rejection” since there is currently no molecular phenotype to these entities. Diagnostic criteria for phenotypes followed the descriptions from: https://cloudfront.ualberta.ca/-/media/medicine/institutes-centres-groups/atagc/report_description.docx.

### Donor-specific antibodies

The presence of donor-specific antibodies (DSAs) was assessed by OneLambda single antigen beads. An adjusted mean fluorescence intensity of ≥500 was considered significant for DSA relevance. Preformed (pDSA) and *de novo* DSA (dnDSA) were analyzed. All individuals received full re-typing for relevant HLA-Antigens (including HLA-C, HLA-DQ, HLA-DP) when needed for interpretation of HLA antibodies occurring in the HLA-Luminex Singles class I or II (DSA vs non-DSA-HLA antibodies).

### Statistical analysis

Statistical analysis was conducted with IBM SPSS Version 29 (SPSS, Chicago, IL, USA), Microsoft^®^ Excel^®^ (©2010 Microsoft Corporation; all rights reserved) and GraphPad Prism Version 9.5.1. Non-parametric tests such as the Mann–Whitney U or Kruskal–Wallis test were performed to compare continuous variables. Fisher's exact or the chi-squared test (χ^2^) were used to compare categorical variables, as appropriate. Probability values and confidence intervals were two-sided. Continuous variables were described using the median and interquartile range (IQR; Q1–Q3) regardless of distribution. Categorical variables were presented as numbers (*n*) and percentages (%). A *P*-value of <.05 was considered significant.

## RESULTS

### Basic and biopsy characteristics

Table 1 displays basic and biopsy characteristics for all groups—additional characteristics (including DSA characteristics) are presented in Supplementary data, Table S1. A greater proportion of isolated arteritis (59.5%) underwent biopsy within the first year post-transplantation (<1 year), compared with the other subgroups in the study cohort (*P* < .001). However, this difference did not significantly affect molecular rejection rates when comparing biopsies conducted <1 year and those after the first year post-transplantation (>1 year) (*P* = .27 for the whole study cohort, *P* = .711 for isolated arteritis; Supplementary data, Fig. S1). Percentages of MVI at/above threshold (14.9%) and subsequent hAMR (11.9%) were significantly lower in no inflammation, compared with TCMR-suspicion (40.8% and 30.6%, respectively) and the positive control cohort (62.9% and 22.9%, respectively; *P* < .001 for MVI and *P* = .03 for hAMR; Supplementary data, Table S2). The whole cohort (*n* = 249) exhibited a high prevalence of DSA at the time of the biopsy (overall 51%).

**Table 1:** Basic and biopsy characteristics.

		Study cohort				
(a)	Study cohort (*n* = 214)	Isolated tubulitis (*n* = 101)	Borderline changes (*n* = 9)	Isolated arteritis (*n* = 37)	No inflammation (*n* = 67)	*P*-value
Female, *n* (%)	83 (38.8)	32 (31.7)	4 (44.4)	20 (54.1)	27 (40.3)	.113
Age at TPL (years)	45 (35–56)	45 (32–56)	42 (29–53)	50 (39–59)	45 (36–56)	.536
Age at Bx (years)	53 (43–62)	52 (42–62)	48 (42–57)	51 (45–61)	57 (46–64)	.376
Deceased donation, *n* (%)	133 (62.1)	61 (60.4)	5 (55.6)	20 (54.1)	47 (70.1)	.369
Repeat TPL, *n* (%)	18 (8.4)	9 (8.9)	0 (0)	4 (10.8)	5 (7.5)	.75
Bx <1 year TPL, *n* (%)	67 (31.3)	22 (21.8)	1 (11.1)	22 (59.5)	22 (32.8)	**<.001**
Bx indication, *n* (%)^a^						
Decrease in eGFR	105 (49.1)	49 (48.5)	6 (66.7)	22 (59.5)	28 (41.8)	.246
Rise in proteinuria	64 (29.9)	26 (25.7)	3 (33.3)	11 (29.7)	24 (35.8)	.571
DSA presence^a^	111 (51.9)	60 (59.4)	5 (55.6)	17 (45.9)	29 (43.3)	.183
Histologic findings, *n* (%)						
MVI below threshold^b^	38 (17.8)	13 (12.9)	1 (11.1)	7 (18.9)	17 (25.4)	.202
MVI at/above threshold^b^	70 (32.7)	36 (35.6)	5 (55.6)	19 (51.4)	10 (14.9)	**<.001**
DSA–, C4d– MVI	21 (9.8)	8 (7.9)	2 (22.2)	9 (24.3)	2 (3)	**.003**
Probable hAMR	57 (26.6)	28 (27.7)	2 (22.2)	7 (18.9)	20 (29.9)	.652
hAMR	53 (24.8)	31 (30.7)	3 (33.3)	11 (29.7)	8 (11.9)	**.034**
Without chronicity	14 (6.5)	7 (6.9)	1 (11.1)	4 (10.8)	2 (3)	.420
DSA+, C4d–	11 (5.1)	5 (5)	1 (11.1)	3 (8.1)	2 (3)	.578
DSA+, C4d+	2 (0.9)	2 (2)	0 (0)	0 (0)	0 (0)	.520
DSA–, C4d+	1 (0.5)	0 (0)	0 (0)	1 (2.7)	0 (0)	.187
cAMR	39 (18.2)	24 (23.8)	2 (22.2)	7 (18.9)	6 (9)	.109
DSA+, C4d–	32 (15)	19 (18.8)	2 (22.2)	5 (13.5)	6 (9)	.199
DSA+, C4d+	7 (3.3)	5 (5)	0 (0)	2 (5.4)	0 (0)	.261
DSA–, C4d+	0 (0)	0 (0)	0 (0)	0 (0)	0 (0)	NA
		**Positive control cohort**				
**(b)**	**Positive control cohort (*n* = 35)**	**TCMR IA/IB (*n* = 9)**	**TCMR IIA/IIB (*n* = 18)**	**Mixed histologic rejection (*n* = 8)**	***P-*value**	
Female, *n* (%)	10 (28.6)	2 (22.2)	6 (33.3)	2 (25)	.807	
Age at TPL (years)	39 (27–53)	38 (33–50)	50 (31–55)	26 (15–32)	**.03**	
Age at Bx (years)	42 (31–56)	40 (36–56)	55 (37–62)	31 (24–41)	.09	
Deceased donation, *n* (%)	21 (60)	6 (66.7)	9 (50.0)	6 (75)	.435	
Repeat TPL, *n* (%)	1 (2.9)	1 (11.1)	0 (0.0)	0 (0)	.226	
Bx <1 year TPL, *n* (%)	17 (48.6)	4 (44.4)	11 (61.1)	2 (25)	.226	
Bx indication, *n* (%)^a^						
Decrease in eGFR	29 (82.9)	7 (77.8)	16 (88.9)	6 (75)	.615	
Rise in proteinuria	10 (28.6)	1 (11.1)	8 (44.4)	1 (12.5)	.101	
DSA presence^a^	16 (45.7)	3 (33.3)	5 (27.8)	8 (100)	**.002**	
Histologic findings, *n* (%)						
MVI below threshold^b^	8 (22.9)	1 (11.1)	7 (38.9)	0 (0)	.058	
MVI at/above threshold^b^	22 (62.9)	5 (55.6)	9 (50.0)	8 (100)	**.045**	
DSA–, C4d– MVI	14 (40)	5 (55.6)	9 (50.0)	0 (0)	1*	
Probable hAMR	4 (11.4)	1 (11.1)	3 (16.7)	0 (0)	1*	
hAMR	8 (22.9)	0 (0)	0 (0)	8 (100)	NA	
Without chronicity	6 (17.1)	0 (0)	0 (0)	6 (75)	NA	
DSA+, C4d–	4 (11.4)	0 (0)	0 (0)	4 (50)	NA	
DSA+, C4d+	2 (5.7)	0 (0)	0 (0)	2 (25)	NA	
DSA–, C4d+	0 (0)	0 (0)	0 (0)	0 (0.0)	NA	
cAMR	2 (5.7)	0 (0)	0 (0)	2 (25)	NA	
DSA+, C4d–	1 (2.9)	0 (0)	0 (0)	1 (12.5)	NA	
DSA+, C4d+	1 (2.9)	0 (0)	0 (0)	1 (12.5)	NA	
DSA–, C4d+	0 (0)	0 (0)	0 (0)	0 (0)	NA	

Part (a) demonstrates the study cohort (*n* = 214) with its subgroups; (b) demonstrates the positive control cohort (*n* = 35) with its subgroups.

Continuous variables are presented as median (IQR, Q1–Q3), categorical variables as numbers (*n*) and percentages (of the underlying group, %).

*Fisher's exact test between TCMR IA/IB and TCMR IIA/IIB. More detailed basic and biopsy characteristics are demonstrated in the Supplementary data.

^a^One patient could have more than one biopsy indication (combinations possible). DSA presence describes the presence of any DSA (pDSA or dnDSA) with relevant mean fluorescence intensity during biopsy.

^b^All biopsies with MVI included (also if satisfying the diagnosis of hAMR).

TPL, transplantation; Bx, biopsy; <1 year, in the first year after transplantation; cAMR, chronic antibody-mediated rejection; NA, not applicable.

### Molecular rejections and histologic differentiation of all biopsies

**Figure 2:**
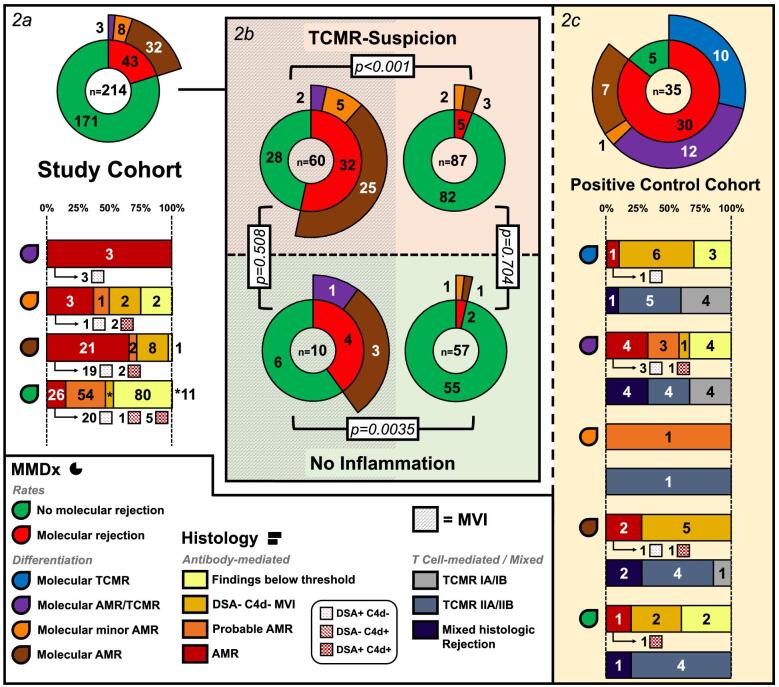
Molecular rejections and histological differentiation—an overview. (**a**) The rates and differentiation of molecular rejections (ring diagram) in the study cohort with the according histologic differentiation (bar charts) and DSA/C4d presence (for cases with hAMR). (**b**) Molecular rejection rates and differentiation of TCMR-suspicion vs no inflammation based on the presence (left part) and absence (right part) of MVI (*P*-values with Fisher's exact test). (**c**) The rates and differentiation of molecular rejections (ring diagram) in the positive control cohort and the histologic differentiation (bar charts).

The rates and differentiation of all molecular rejections are demonstrated as an overview in Table 2 and Fig. 2 and with more histology details in Supplementary data, Table S3. Molecular rejection rates were 37/147 (25.2%; 32 with MVI) in TCMR-suspicion, 6/67 (9%; 4 with MVI) in no inflammation and 30/35 (85.7%; 19 with MVI) in the positive control cohort. As visible in Fig. 2b, 32/60 cases (53.3%) with TCMR-suspicion and MVI showed molecular rejections (2 mAMR/TCMR, 5 minor mAMR and 25 mAMR) compared with 4/10 cases (40%) with no inflammation but MVI (1 mAMR/TCMR and 3 mAMR; *P* = .508). However, 5/87 cases (5.7%) only with TCMR-suspicion but MVI below threshold/negative showed molecular rejections (2 minor mAMR and 3 mAMR) compared with 2/57 cases (3.5%) with no inflammation and MVI below threshold/negative (1 minor mAMR and 1 mAMR; *P* = .704). Twenty-two of 32 (68.9%) molecular rejection cases with TCMR-suspicion and MVI had hAMR (31.1% did not), compared with 4/4 cases (100%) with no inflammation but MVI (Supplementary data, Table S4). Only 3 mAMR/TCMR (1.4%) and no pure mTCMR were detected in the study cohort, compared with 12 mAMR/TCMR (34.3%) and 10 mTCMR (28.6%) in the positive control cohort (*P* < .001). Molecular rejection rates in the positive control cohort were similar when subdivided based on the presence/absence of MVI; however, all cases of pure mAMR in this group (*n* = 7) had MVI at/above threshold (Supplementary data, Fig. S2 and Table S3). When subdividing the positive control cohort based on TCMR1 or TCMR2 phenotypes, all pure mTCMR-cases (*n* = 10) were among TCMR2, while TCMR1 cases exhibited non-significantly, but slightly more mAMR (33.3%) and mAMR/TCMR (38.9%) than TCMR2 (5.9% and 29.4%; *P* = .088 and *P* = .725, respectively; Supplementary data, Table S5).

**Table 2:** Molecular phenotypes in all subgroups.

		Study cohort			
(a)	Study cohort (*n* = 214)	Isolated tubulitis (*n* = 101)	Borderline changes (*n* = 9)	Isolated arteritis (*n* = 37)	No inflammation (*n* = 67)
Molecular rejection, *n* (%)	43 (20.1)	23 (22.8)	5 (55.6)	9 (24.3)	6 (9)
mTCMR	0 (0)	0 (0)	0 (0)	0 (0)	0 (0)
mAMR/TCMR	3 (1.4)	1 (1)	1 (11.1)	0 (0)	1 (1.5)
Minor mAMR	8 (3.7)	6 (5.9)	0 (0)	1 (2.7)	1 (1.5)
mAMR	32 (15)	16 (15.8)	4 (44.4)	8 (21.6)	4 (6)
No molecular rejection, *n* (%)	171 (79.9)	78 (77.2)	4 (44.4)	28 (75.7)	61 (91)
		**Positive control cohort**			
**(b)**	**Positivecontrolcohort(*n* = 35)**	**TCMR IA/IB(*n* = 9)**	**TCMR IIA/IIB(*n* = 18)**	**Mixedhistologic rejection(*n* = 8)**	
Molecular rejection, *n* (%)	30 (85.7)	9 (100)	14 (77.8)	7 (87.5)	
mTCMR	10 (28.6)	4 (44.4)	5 (27.8)	1 (12.5)	
mAMR/TCMR	12 (34.3)	4 (44.4)	4 (22.2)	4 (50)	
Minor mAMR	1 (2.9)	0 (0)	1 (5.6)	0 (0)	
mAMR	7 (20)	1 (11.1)	4 (22.2)	2 (25)	
No molecular rejection, *n* (%)	5 (14.3)	0 (0)	4 (22.2)	1 (12.5)	

Part (a) demonstrates the study cohort (*n* = 214) with its subgroups; (b) demonstrates the positive control cohort (*n* = 35) with its subgroups.

Categorical variables as numbers and percentages (of the underlying group, %).

### Molecular rejections and histologic differentiation among subgroups of TCMR-suspicion

**Figure 3:**
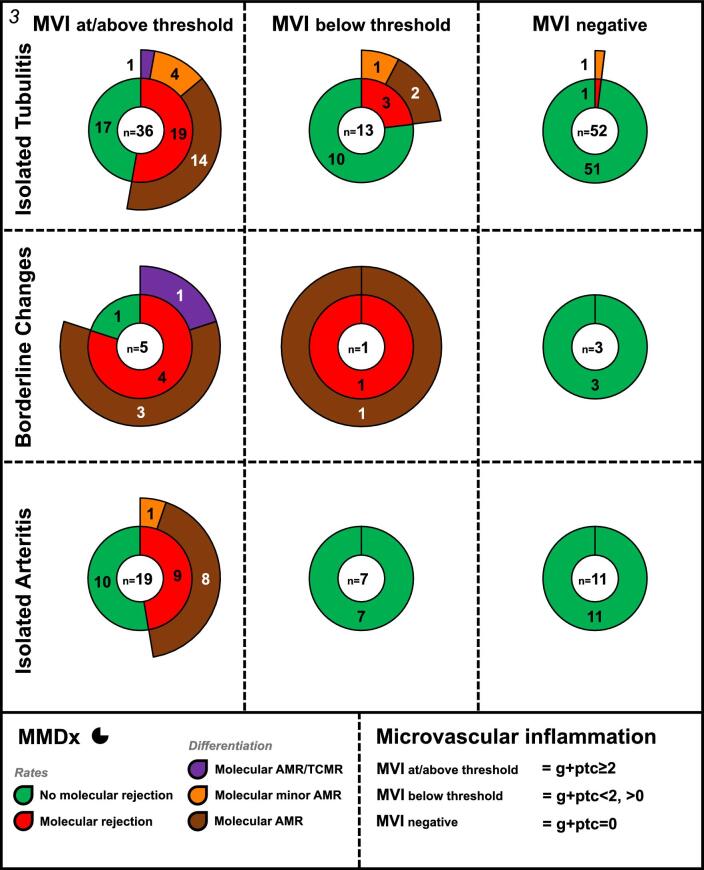
Molecular rejection rates and differentiation—subgroups of TCMR-suspicion: molecular rejection rates and differentiation for the subgroups are separately presented based on the presence of MVI at/above threshold (left), MVI below threshold (middle) and no MVI (right). Overall, 32/37 (86.5%) molecular rejections are associated with MVI at/above threshold and only 5/37 (13.5%) with MVI below threshold/negative.

The highest molecular rejection rate was observed in borderline changes with MVI (80%), followed by isolated tubulitis with MVI (52.8%; Fig. 3). Rejection rates were significantly lower if MVI was below threshold or negative (*P* < .001 for all groups with MVI at/above threshold vs below threshold/negative).

### Molecular (Classifier) and rejection phenotype scores for TCMR

**Figure 4:**
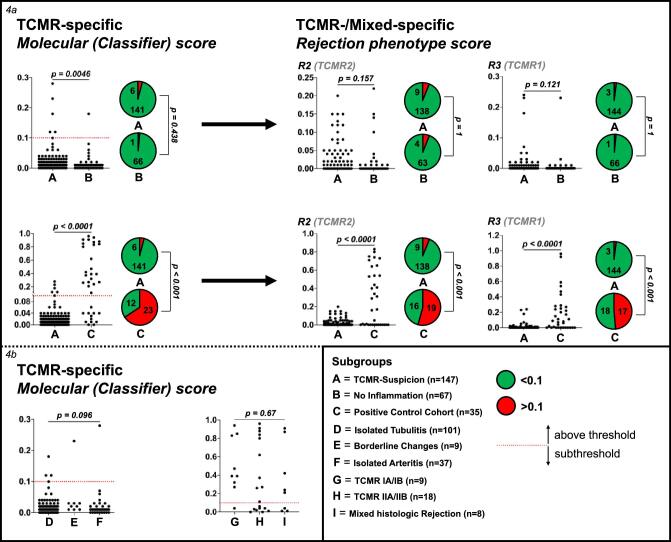
Molecular (Classifier) and rejection phenotype scores for TCMR (R2 and R3). (**a**) The comparison of the TCMR-specific molecular (Classifier) score (left of the arrow) and rejection phenotype scores R2 or R3 (right of the arrow) in TCMR-suspicion (A), no inflammation (B) and the positive control cohort (C). Also, the comparison of the TCMR-specific molecular score in between the subgroups of TCMR-suspicion (D–F) and the positive control cohort (G–I) is demonstrated (**b**). Pie charts demonstrate the values (*n*) ≥0.1 in groups A and B or C (*P*-values for comparison with Fisher's exact test). In the study cohort (*n* = 214), four cases with subthreshold TCMR-specific findings [probable TCMR (pTCMR), as defined by the establishing group in Alberta, Canada in: https://cloudfront.ualberta.ca/-/media/medicine/institutes-centres-groups/atagc/report_description.docx] were found, which corresponded to two mAMR/TCMR cases (one from borderline changes and one from no inflammation), one mAMR (from isolated tubulitis) and one no molecular rejection (from isolated arteritis).

TCMR-specific molecular (Classifier) scores were different between TCMR-suspicion (median 0.010, IQR 0.010–0.020) and no inflammation (median 0.010, IQR 0.000–0.010; *P* = .005) and between TCMR-suspicion and the positive control cohort (median 0.27, IQR 0.040–0.83; *P* < .001; Fig. 4). However, only 6/147 (4.1%) cases from TCMR-suspicion and 1/67 (1.5%) cases from no inflammation showed a TCMR-specific Classifier score ≥0.1 (*P* = .438). As expected, the number of cases with a TCMR-specific molecular (Classifier) score ≥0.1 was significantly higher in the positive control cohort (23/35, 65.7%; *P* < .001 vs TCMR-suspicion). Looking at rejection phenotype scores relevant for mTCMR or mAMR/TCMR (R2 and R3), there was no differentiation between TCMR-suspicion and no inflammation (*P* = .157 for R2 and 0.121 for R3), but a clear differentiation between TCMR-suspicion and the positive control cohort overall, and above a score of 0.1 (*P* < .001 for all comparisons). Subthreshold findings, suggested as probable TCMR (pTCMR) with TCMR-specific molecular scores >0.1 and R2 + R3 >0.2 but not fulfilling mTCMR criteria were scarce (*n* = 4) and corresponded to mAMR/TCMR in two cases, mAMR in one case and no molecular rejection in one case. Detailed MMDx scores in all subgroups are described in Table 3.

**Table 3:** MMDx scores in all subgroups.

		Study cohort				
(a)	Study cohort (*n* = 214)	Isolated tubulitis (*n* = 101)	Borderline changes (*n* = 9)	Isolated arteritis (*n* = 37)	No inflammation (*n* = 67)	*P-*value
% Cortex (%)	85 (71–91)	85 (74–91)	88 (49–93)	83 (73–92)	85 (61–90)	.841
R scores						
R1 (no rejection)	0.80 (0.60–0.90)	0.77 (0.56–0.89)	0.11 (0.00–0.77)	0.71 (0.52–0.87)	0.87 (0.79–0.91)	**<.001**
R2 (TCMR)	0.00 (0.00–0.01)	0.00 (0.00–0.01)	0.00 (0.00–0.02)	0.00 (0.00–0.01)	0.00 (0.00–0.00)	.543
R3 (mixed)	0.00 (0.00–0.00)	0.00 (0.00–0.00)	0.01 (0.00–0.02)	0.00 (0.00–0.00)	0.00 (0.00–0.00)	**.004**
R4 (early AMR)	0.05 (0.00–0.15)	0.05 (0.00–0.17)	0.23 (0.04–0.40)	0.08 (0.02–0.28)	0.04 (0.00–0.08)	**.01**
R5 (full AMR)	0.01 (0.00–0.06)	0.02 (0.00–0.09)	0.10 (0.02–0.50)	0.01 (0.00–0.11)	0.00 (0.00–0.02)	**<.001**
R6 (late AMR)	0.05 (0.00–0.14)	0.04 (0.01–0.15)	0.08 (0.00–0.21)	0.04 (0.01–0.18)	0.05 (0.00–0.11)	.978
R7 (all AMR)	0.18 (0.09–0.38)	0.22 (0.10–0.45)	0.73 (0.21–0.99)	0.26 (0.12–0.48)	0.12 (0.08–0.20)	**<.001**
R8 (all TCMR)	0.00 (0.00–0.01)	0.00 (0.00–0.02)	0.02 (0.00–0.03)	0.00 (0.00–0.02)	0.00 (0.00–0.01)	**.039**
Mol. injury scores						
Global disturbance	−1.56 (−2.92–0.25)	−1.53 (−2.79–0.36)	−0.19 (−2.13–1.73)	−0.19 (−1.65–1.2)	−2.55 (−3.67 to −1.19)	**<.001**
AKI	−0.08 (−0.48–0.41)	−0.05 (−0.43–0.38)	−0.12 (−0.44–0.56)	0.27 (−0.2–0.7)	−0.35 (−0.64–0.05)	**<.001**
Atrophy fibrosis	0.24 (0.12–0.56)	0.29 (0.14–0.66)	0.4 (0.19–0.73)	0.27 (0.12–0.52)	0.18 (0.1–0.43)	**.03**
Mol. rej. scores						
Rejection_prob_	0.04 (0.02–0.18)	0.07 (0.02–0.22)	0.68 (0.06–0.85)	0.08 (0.03–0.26)	0.02 (0.01–0.04)	**<.001**
TCMR_prob_	0.01 (0.01–0.02)	0.01 (0.01–0.02)	0.02 (0.01–0.03)	0.01 (0.01–0.02)	0.01 (0–0.01)	**.005**
AMR_prob_	0.05 (0.04–0.14)	0.06 (0.04–0.18)	0.29 (0.06–0.88)	0.08 (0.04–0.17)	0.04 (0.03–0.07)	**<.001**
Mol. histol. scores						
g >0_prob_	0.15 (0.09–0.28)	0.16 (0.09–0.33)	0.57 (0.21–0.77)	0.18 (0.14–0.37)	0.11 (0.08–0.16)	**<.001**
cg >0_prob_	0.10 (0.05–0.21)	0.11 (0.06–0.25)	0.21 (0.12–0.52)	0.11 (0.06–0.24)	0.08 (0.04–0.14)	**.004**
ptc >0_prob_	0.10 (0.06–0.22)	0.11 (0.07–0.27)	0.58 (0.11–0.78)	0.13 (0.08–0.34)	0.07 (0.05–0.1)	**<.001**
DSA+_prob_ *n* = 114	0.42 (0.32–0.55)	0.45 (0.31–0.61)	0.79 (0.55–0.81)	0.35 (0.32–0.55)	0.38 (0.31–0.47)	**.003**
i >0_prob_	0.02 (0.01–0.05)	0.02 (0.01–0.05)	0.05 (0.03–0.09)	0.03 (0.01–0.05)	0.02 (0.01–0.02)	**<.001**
t >0_prob_	0.03 (0.02–0.05)	0.04 (0.02–0.06)	0.07 (0.04–0.1)	0.03 (0.02–0.05)	0.03 (0.02–0.04)	**.001**
ct >0_prob_	0.19 (0.10–0.41)	0.22 (0.11–0.5)	0.28 (0.16–0.66)	0.22 (0.1–0.4)	0.13 (0.08–0.31)	.072
ah >0_prob_ *n* = 114	0.55 (0.44–0.68)	0.57 (0.46–0.7)	0.66 (0.55–0.79)	0.55 (0.36–0.62)	0.55 (0.41–0.68)	.253
		**Positive control cohort**				
**(b)**	**Positivecontrolcohort(*n* = 35)**	**TCMR IA-IB(*n* = 9)**	**TCMR IIA-IIB(*n* = 18)**	**Mixedhistologic rejection** **(*n* = 8)**	***P-*value**	
% Cortex (%)	60 (3–81)	42 (1–81)	27 (2–83)	72 (41–93)	.265	
R scores						
R1 (no rejection)	0.00 (0.00–0.11)	0.02 (0.00–0.20)	0.00 (0.00–0.16)	0.00 (0.00–0.05)	.617	
R2 (TCMR)	0.23 (0.00–0.57)	0.40 (0.23–0.69)	0.03 (0.00–0.56)	0.00 (0.00–0.52)	.208	
R3 (mixed)	0.09 (0.00–0.26)	0.22 (0.00–0.27)	0.10 (0.01–0.22)	0.04 (0.00–0.40)	.907	
R4 (early AMR)	0.00 (0.00–0.20)	0.00 (0.00–0.27)	0.00 (0.00–0.18)	0.03 (0.00–0.44)	.76	
R5 (full AMR)	0.06 (0.00–0.27)	0.00 (0.00–0.05)	0.06 (0.00–0.31)	0.11 (0.06–0.50)	**.043**	
R6 (late AMR)	0.00 (0.00–0.14)	0.07 (0.00–0.23)	0.01 (0.00–0.18)	0.00 (0.00–0.00)	.17	
R7 (all AMR)	0.32 (0.05–0.68)	0.22 (0.04–0.52)	0.35 (0.00–0.78)	0.52 (0.09–0.83)	.624	
R8 (all TCMR)	0.44 (0.07–0.94)	0.54 (0.38–0.96)	0.30 (0.04–0.98)	0.44 (0.02–0.83)	.514	
Mol. injury scores						
Global disturbance	4.45 (1.87–6.17)	5.25 (1.63–8.04)	3.87 (1.83–5.86)	5.23 (1.15–8.09)	.417	
AKI	0.89 (0.44–1.59)	0.97 (0.46–1.52)	0.88 (0.25–1.59)	1.03 (0.43–1.61)	.927	
Atrophy fibrosis	0.55 (0.22–0.78)	0.42 (0.17–0.74)	0.50 (0.32–0.76)	0.62 (0.25–0.89)	.671	
Mol. rej. scores						
Rejection_prob_	0.81 (0.41–0.92)	0.64 (0.41–0.82)	0.87 (0.25–0.93)	0.91 (0.63–0.95)	.249	
TCMR_prob_	0.27 (0.04–0.83)	0.39 (0.30–0.84)	0.19 (0.03–0.81)	0.22 (0.02–0.76)	.301	
AMR_prob_	0.34 (0.1–0.8)	0.31 (0.09–0.47)	0.23 (0.11–0.81)	0.61 (0.16–0.90)	.374	
Mol. histol. scores						
g >0_prob_	0.34 (0.17–0.72)	0.17 (0.15–0.48)	0.34 (0.27–0.82)	0.52 (0.32–0.85)	.07	
cg >0_prob_	0.16 (0.08–0.44)	0.14 (0.08–0.36)	0.31 (0.08–0.48)	0.16 (0.07–0.76)	.722	
ptc >0_prob_	0.63 (0.35–0.82)	0.55 (0.31–0.63)	0.55 (0.27–0.83)	0.80 (0.65–0.89)	.139	
DSA+ _prob_ *n* = 17	0.62 (0.40–0.79)	0.31 (0.25–0.71)	0.62 (0.45–0.78)	0.80 (0.43–0.89)	.279	
**(b)**	**Positivecontrolcohort(*n* = 35)**	**TCMR IA-IB(*n* = 9)**	**TCMR IIA-IIB(*n* = 18)**	**Mixedhistologic rejection** **(*n* = 8)**	***P-*value**	
i >0_prob_	0.53 (0.12–0.92)	0.77 (0.37–0.94)	0.50 (0.09–0.93)	0.58 (0.06–0.91)	.659	
t >0_prob_	0.45 (0.15–0.71)	0.47 (0.32–0.75)	0.30 (0.09–0.83)	0.42 (0.07–0.66)	.549	
ct >0_prob_	0.47 (0.22–0.75)	0.35 (0.13–0.69)	0.39 (0.27–0.76)	0.56 (0.20–0.85)	.762	
ah >0_prob_ *n* = 17	0.30 (0.17–0.56)	0.16 (0.10–0.55)	0.32 (0.17–0.57)	0.30 (0.22–0.63)	.464	

Part (a) demonstrates the study cohort (*n* = 214) with its subgroups; (b) demonstrates the positive control cohort (*n* = 35) with its subgroups. Continuous variables are presented as median (IQR, Q1–Q3).

R7 equals to the “all AMR” score (R4 + R5 + R6), R8 signifies the “all TCMR” score, meaning R2 + R3.

Ranges (the 2.5th to 97.5th percentiles in the entire reference set) for molecular injury scores is for global disturbance score –3.8 to 5.8; AKI score –0.6 to 1.6; atrophy fibrosis score 0–1. The upper limit of normal (90th percentile in the relevant reference set biopsies) is not set to one value and differs for each biopsy according to the reference set biopsies.

Ranges (the 2.5th to 97.5th percentiles in the entire reference set) for all molecular rejection scores is 0–1. The upper limit of normal (90th percentile in the relevant reference set biopsies) is for rejection score 0.3; TCMR score 0.1; AMR score 0.2.

R scores, rejection phenotype scores; Mol., molecular; rej., rejection; histol., histology; prob, probability; g, glomerulitis; cg, glomerular basement membrane double contours; ptc, peritubular capillaritis; i, interstitial inflammation; t, tubulitis; ct, tubular atrophy; ah, arteriolar hyalinosis.

### Molecular mixed phenotypes

All mAMR/TCMR cases (*n* = 15) among the whole cohort (*n* = 249) are visible in Table 4. All 3/3 cases (100%, case numbers 1–3) from the study cohort (*n* = 214) had MVI/hAMR compared with only 3/12 cases (25%) and 4/12 cases (33.3%) from the positive control cohort (case numbers 4–15), respectively. R score patterns showed heterogeneity with higher all AMR (R7 >50%) and lower all TCMR (R8 <50%) scores in case numbers 1–3 compared with R scores in case numbers 4–15 (visualized in Supplementary data, Fig. S3), yet, ultimately leading to the same molecular phenotype.

**Table 4:** Mixed molecular cases (mAMR/TCMR cases) in detail.

	Cohort	Histology									Serology	Molecular scores		R scores			
Case Nr	Group	i/t/v	MVI	ti	i-IFTA	C4d+	cg	cv	ci	ct	DSA	TCMR	AMR	R2	R3	R7	R8
1	SC (IT)	i0, t1, v0	g3, ptc2	0	2	No	2	1	0	1	dnDSA	0.18	0.57	0	0.18	0.81	0.18
2	SC (BL)	i1, t1, v0	g1, ptc2	2	3	No	1b	0	2	2	dnDSA	0.23	0.58	0.03	0.23	0.73	0.26
3	SC (NI)	i0, t0, v0	g2, ptc2	3	3	No	0	0	3	3	p + dnDSA	0.18	0.94	0.04	0.23	0.73	0.27
4	PCC (M)	i2, t3, v0	g1, ptc2	2	0	No	0	0	1	1	dnDSA	0.42	0.39	0.54	0	0.46	0.54
5	PCC (T)	i3, t3, v0	g0, ptc0	3	0	No	0	0	0	0	negative	0.85	0.31	0.74	0.24	0	0.98
6	PCC (T)	i2, t3, v0	g0, ptc2	2	3	No	0	0	1	1	dnDSA	0.47	0.42	0.16	0.28	0.55	0.44
7	PCC (T)	i2, t3, v0	g0, ptc0	2	3	No	0	1	1	1	pDSA	0.83	0.51	0.4	0.54	0.07	0.94
8	PCC (T)	i3, t3, v0	g1, ptc1	3	3	No	1a	1	3	3	negative	0.32	0.39	0.29	0.22	0.48	0.51
9	PCC (T)	i3, t2, v1	g0, ptc0	3	0	No	0	1	0	0	dnDSA	0.88	0.34	0.73	0.27	0	1
10	PCC (T)	i3, t3, v1	g0, ptc2	3	3	No	0	1	1	1	pDSA	0.96	0.58	0.41	0.59	0	1
11	PCC (M)	i1, t2, v1	g1, ptc1	1	1	No	0	1	2	2	p + dnDSA	0.24	0.38	0.44	0	0.57	0.44
12	PCC (T)	i3, t2, v1	gX, ptc2	3	3	No	X	1	2	2	dnDSA	0.26	0.91	0	0.47	0.53	0.47
13	PCC (M)	i2, t3, v1	g1, ptc2	3	3	Yes	0	3	2	2	dnDSA	0.91	0.91	0	0.92	0.08	0.92
14	PCC (M)	i1, t1, v2	g2, ptc2	1	3	No	0	1	1	1	p + dnDSA	0.21	0.96	0	0.43	0.57	0.43
15	PCC (T)	i2, t3, v2	g0, ptc1	2	3	No	0	0	1	1	negative	0.91	0.89	0.01	0.96	0	0.97

R7 equals the “all AMR” score (R4 + R5 + R6), R8 signifies the “all TCMR” score, meaning R2 + R3.

All three cases of mAMR/TCMR from within the study cohort (SC) are presented (Case Nr 1–3), as well as the 12 cases of mAMR/TCMR from the positive control cohort (PCC; case Nr 4–15). The most important Banff lesions and DSA/C4d status, as well as the relevant molecular (Classifier) scores and rejection phenotype scores (R scores) are demonstrated in detail.

SC (IT), isolated tubulitis; SC (BL), borderline changes; SC (NI), no inflammation; PCC (M), hAMR/TCMR; PCC (T), hTCMR; Nr, number; IFTA, interstitial fibrosis and tubular atrophy; pDSA, preformed DSA; dnDSA, *de novo* DSA; SC, study cohort; PCC, positive control cohort.

### Patterns of Banff lesions and DSA among all molecular rejections

**Figure 5:**
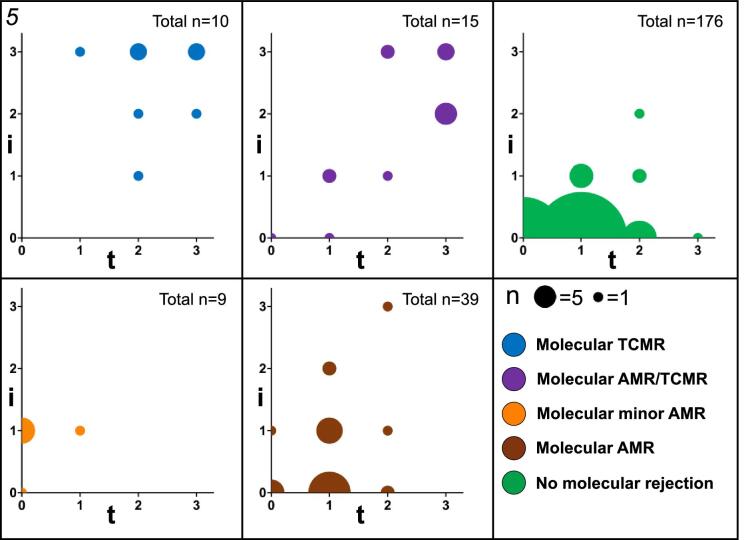
Plotting of all molecular rejections according to Banff i- and t-lesions: all molecular rejections (*n* = 73) from the whole cohort are divided according to their phenotype (see legend with different colors). The y-axis represents semiquantitative i-lesions, while the x-axis represents t-lesions based on histology. The size of the bubbles represents the number (*n*) of cases. mTCMR cases occur mostly with higher degrees of inflammation (i + t), whereas mAMR cases also occur in lower inflammation based on i- and t-lesions.

Throughout all molecular rejections from the whole cohort (73/249, 29.3%), 8/10 cases (80%) with mTCMR exhibited i >1 and t >1 by histology compared with 10/15 cases (66.7%) of mAMR/TCMR and only 1/39 cases (2.6%) of mAMR (*P* < .001; Table 5 and Fig. 5). v >0 was not significantly different between the different molecular rejection groups (*P* = .551). However, presence of MVI at/above threshold was highest in mAMR (35/39, 89.7%). DSA presence (pDSA or dnDSA) was 30% in mTCMR, 80% in mAMR/TCMR, 55.6% in minor mAMR and 61.5% mAMR (*P* = .085, Table 5). The time point of first detection of dnDSA was significantly earlier in mTCMR and mAMR/TCMR compared with minor mAMR or mAMR (*P* < .001; also Table 5). Detailed individual Banff lesions for different subgroups are demonstrated in Supplementary data, Table S6.

**Table 5:** Banff lesion patterns and DSA presence in all molecular rejections.

	All molecular rejections from the whole cohort				
	mTCMR (*n* = 10)	mAMR/TCMR (*n* = 15)	Minor mAMR (*n* = 9)	mAMR (*n* = 39)	*P-*values
T-cell-related changes, *n* (%)					
i >0	10 (100)	13 (86.7)	1 (11.1)	12 (30.8)	**<.001**
t >0	10 (100)	14 (93.3)	8 (88.9)	31 (79.5)	.284
i >0 and t >0	10 (100)	13 (86.7)	1 (11.1)	11 (28.2)	**<.001**
i >1 and t >1	8 (80)	10 (66.7)	0 (0)	1 (2.6)	**<.001**
v >0	5 (50)	7 (46.7)	2 (22.2)	14 (35.9)	.551
v >0 and i + t >1	5 (50)	7 (46.7)	1 (11.1)	6 (15.4)	**.022**
v >0 and i + t >2	5 (50)	6 (40)	0 (0)	3 (7.7)	**.001**
Antibody-related changes					
MVI at/above threshold, *n* (%)^a^	7 (70)	8 (53.3)	5 (55.6)	35 (89.7)	**.016**
C4d-positivity, *n* (%)	0 (0)	1 (6.7)	2 (22.2)	3 (7.7)	.348
Presence of DSA (yes), *n* (%)	3 (30)	12 (80)	5 (55.6)	24 (61.5)	.947
pDSA	0 (0)	5 (33.3)	2 (22.2)	7 (17.9)	.221
dnDSA	3 (30)	10 (66.7)	4 (44.4)	17 (43.6)	.296
dnDSA detection to biopsy (days)	1 (0)	112 (19–235)	1824 (1632–2121)	1675 (294–2561)	**<.001**

Continuous variables are presented as median (IQR, Q1–Q3), categorical variables as numbers and percentages (of the underlying group, %).

^a^All biopsies with MVI included (also if satisfying the diagnosis of hAMR).

pDSA, preformed DSA; dnDSA, *de novo* DSA.

## DISCUSSION

Transplant physicians hope that MMDx will help to distinguish TCMR presence or absence in vague histologic findings of i-, t- or v-lesions below the threshold of hTCMR. Such a biomarker is crucial since intensifying immunosuppression in these vulnerable patients causes morbidity (e.g. infections) [33]. In contrast, the presence of even subclinical hTCMR is associated with an increased risk of developing alloimmunity, interstitial fibrosis and tubular atrophy, and reduced graft survival [34]. In our biopsy cohort we observed that MMDx does not identify pure mTCMR along the continuum of TCMR-suspicion regardless of MVI presence or absence.

However, MMDx differentiates between mAMR/TCMR and pure mAMR in the patient cohort with TCMR-suspicous lesions and the presence of MVI. All three mAMR/TCMR cases within the study cohort fulfilled Banff criteria for hAMR. All other molecular rejections in the study cohort were mAMR (either minor or full), with MVI as the driving force. A recent study investigating a transitional B-cell-based risk stratifying biomarker in the setting of borderline rejection suggested that especially cases with borderline changes in combination with moderate MVI (g+ptc ≥2) had poorer long-term outcomes, but the graft survival in individuals without MVI was similar to KTRs without suspicious findings in compared surveillance kidney biopsies [35, 36]. In our study, the occurrence of MVI in all TCMR-suspicion subgroups was strongly associated with mAMR but not mTCMR, suggesting a microvascular disease that usually spares other components of the parenchyma. Therefore, MMDx in TCMR-suspicion may be useful to identify concomitant mAMR or mAMR/TCMR, particularly since Banff 2022 maintains to discount isolated ptc in the presence of tubulitis or borderline changes [32]. Four patients in our cohort showed g0, ptc2 with at least borderline (i and t) lesions, from which three had mAMR/TCMR. Furthermore, almost one-third of biopsies with either minor mAMR, mAMR or mAMR/TCMR in the group with TCMR-suspicion and MVI did not fulfill hAMR criteria. While MMDx can reclassify biopsies with histologic suspicion of AMR to mAMR, it does not provide the same added diagnostic benefit in the TCMR continuum as presented in earlier studies. Reeve *et al*. even observed a lower rate of mTCMR in cases of hTCMR [37]. The agreement between pathologists in our hospital and MMDx was comparable (28.6% for mTCMR and 34.3% mAMR/TCMR in the positive control cohort). One possible explanation for this phenomenon is that MVI takes precedence over lower levels of i- and t-lesions in the diagnosis of mAMR or mAMR/TCMR. This observation was consistent with the findings of the current study, where cases of mTCMR in the positive control cohort consistently exhibited significant i + t-scores (sum >2). Additionally, all calls of mAMR within the positive control cohort (*n* = 7) were associated with MVI at/above threshold. This underlines the opinion of many experts that MMDx sets the threshold to diagnose mTCMR—or to detect TCMR-specific lesions as relevant—too high, whereas the threshold for mAMR is considerably lower.

While the TCMR-specific molecular score differentiated between TCMR-suspicion and no inflammation on subthreshold levels, the R score relevant for mTCMR (R2) did not. R2 scores higher than 0.1 in this study (*n* = 13) corresponded to mAMR in two cases and no molecular rejection in 11 cases. Regardless of subthreshold differentiation, the Classifier activity seemed to be too low to generate a mTCMR phenotype. Beyond this generally low sensitivity for TCMR-specific lesions, diversion from other histologic findings, such as infections or the recurrence of a primary disease, could be an explanation. However, we excluded cases with overlapping pathological findings (e.g. BK nephropathy) from the study cohort since minor molecular findings (normal rejection but abnormal TCMR score or vice versa) were observed, especially in overlapping pathologic findings (data not shown).

mTCMR has been recently differentiated into two TCMR classes. TCMR1 shows higher mTCMR activity and mAMR activity (mixed rejection), including v-lesions, and TCMR2 shows less mTCMR activity but more atrophy fibrosis [34]. Interestingly, in this work, the authors show only 12 cases out of a total cohort of 1679 cases (INTERCOMEX study) with borderline changes by histology and mTCMR, with 2 cases attributed to TCMR1 (formerly mixed) and 10 cases to TCMR2. However, in this very small group of 10 cases with borderline changes and TCMR2, it remains unclear how many actually had MVI (18% of cases with TCMR2 showed mAMR/TCMR) or fulfilled the histologic criteria for caTCMR (although a debated entity) because of the higher proportion of atrophy fibrosis. Also, based on the data presented by the INTERCOMEX study, it remains open to what extent confounding diseases were present. In our study we found TCMR1 cases consisted of more mAMR and mAMR/TCMR whereas pure mTCMR phenotypes were only observed in the TCMR2 subgroup.

Of course, the accuracy of MMDx is not guaranteed—discrepancy rates for TCMR and AMR detection (histology vs molecular diagnostics) are known [25, 38]. In this respect, our data do not allow conclusions on whether the molecular diagnosis is correct or incorrect in our cohort. However, a biomarker that does not differentiate in a cohort with TCMR-suspicion does not ultimately enhance clinical decision-making. In this context, however, it can be assumed that the interobserver variation in histology assessment is also reflected. We therefore recommend that transplant centers with high rates of borderline changes investigate their individual added value of MMDx in TCMR-suspicion. While the study cohort had excellent data quality and substantial biopsy numbers, it has some limitations. The histopathological diagnosis was made by specialized pathologists from the University Hospital of Zurich without a second opinion. Also, pending patient follow-up data is crucial for retrospectively assessing the impact of MMDx interpretation, anti-rejection treatment, kidney function and alloimmunity development. Follow-up biopsies are needed to further assess the clinical relevance of our findings.

## CONCLUSION

After thorough exclusion of overlapping pathological findings, MMDx did not identify pure mTCMR in patients with isolated tubulitis, borderline changes or isolated arteritis, regardless of MVI, and identified mAMR and mAMR/TCMR more frequently. The rate of mTCMR was also low in the positive control cohort, consisting of hTCMR and hAMR/TCMR. Our data suggest that MMDx has a lower sensitivity for TCMR lesions compared with AMR lesions, but may be useful in identifying concomitant mAMR or ruling out molecular TCMR activity in suspicious cases with subthreshold activity. Future studies with targeted interventions and follow-up biopsies should investigate whether this differentiation affects graft outcomes.

## Supplementary Material

gfae147_Supplemental_File

## Data Availability

The data that support the findings of this study are available on request from the corresponding author, T.S. The data are not publicly available due to restrictions, e.g. their containing information that could compromise the privacy of research participants.
