# Supplementary material for: Limitations of biopsy-based transcript diagnostics to detect T-cell-mediated allograft rejection
Source: Nephrol Dial Transplant. 2024 Jun 26;40(2):294–307. doi: 10.1093/ndt/gfae147 (PMC11852332; doi:10.1093/ndt/gfae147)
Supplement: gfae147_Supplemental_File [file gfae147_Supplemental_File.pdf]

SUPPLEMENTARY MATERIALS

Supplementary Tables

1. Supplementary Table 1: Additional basic and biopsy characteristics
2. Supplementary Table 2: Basic, biopsy and MMDx characteristics between TCMR-Suspicion and No Inflammation
3. Supplementary Table 3: Histology details of molecular phenotypes in subgroups
4. Supplementary Table 4: Correlation of molecular rejections with hAMR among subgroups
5. Supplementary Table 5: Subgroups of TCMR1 and TCMR2 among the Positive Control Cohort
6. Supplementary Table 6: Detailed Banff-lesions of all subgroups
7. Supplementary Table 7: Molecular rejection rates and differentiation – subgroups of biopsy indications

Supplementary Figures

1. Supplementary Figure 1: Molecular rejection rates and differentiation – subgroups of time point
2. Supplementary Figure 2: Molecular rejection rates and differentiation – Positive Control Cohort with/without MVI
3. Supplementary Figure 3: Rejection phenotype scores of different subgroups

Supplementary Tables

a)	Study Cohort (n=214)	Isolated Tubulitis (n=101)	Borderline Changes (n=9)	Isolated Arteritis (n=37)	No Inflammation (n=67)	*p-values*
**Immunosuppression**						
Tacrolimus n (%)	151 (70.6)	75 (74.3)	7 (77.8)	27 (73)	42 (62.7)	*0.393*
Ciclosporin n (%)	15 (7)	6 (5.9)	1 (11.1)	1 (2.7)	7 (10.4)	*0.444*
Mycophenolate n (%)	172 (80.4)	86 (85.1)	7 (77.8)	26 (70.3)	53 (79.1)	*0.266*
Azathioprin n (%)	24 (11.2)	10 (9.9)	2 (22.2)	6 (16.2)	6 (9)	*0.468*
Everolimus/Sirolimus n (%)	9 (4.2)	4 (4)	0 (0)	4 (10.8)	1 (1.5)	*0.13*
Belatacept n (%)	39 (18.2)	18 (17.8)	1 (11.1)	7 (18.9)	13 (19.4)	*0.942*
Prednisone n (%)	170 (79.4)	87 (86.1)	8 (88.9)	31 (83.8)	44 (65.7)	***0.009***
**Bx after TPL (y)**	3 (0-12)	4 (1-11.5)	3 (1.5-14)	0 (0-9)	3 (0-16)	***0.04***
**Presence of DSA $**						
pDSA n (%)	32 (15)	15 (14.9)	2 (22.2)	3 (8.1)	12 (17.9)	*0.532*
HLA Class I n (%)	15 (7)	8 (7.9)	1 (11.1)	2 (5.4)	4 (6)	*0.892*
HLA Class II n (%)	27 (12.6)	14 (13.9)	2 (22.2)	2 (5.4)	9 (13.4)	*0.443*
dnDSA n (%)	86 (40.2)	50 (49.5)	3 (33.3)	14 (37.8)	19 (28.4)	*0.05*
HLA Class I n (%)	23 (10.7)	11 (10.9)	2 (22.2)	6 (16.2)	4 (6)	*0.263*
HLA Class II n (%)	73 (34.1)	42 (41.6)	2 (22.2)	12 (32.4)	17 (25.4)	*0.145*
**Donor age at TPL (y)**	49 (41-58)	48 (41-57)	56 (47-63)	50 (45-63)	47 (39-59)	*0.212*
**Donor sex**						*0.959**
Female n (%)	101 (47.2)	50 (49.5)	6 (66.7)	21 (56.8)	24 (35.8)	
Male (n%)	105 (49.1)	46 (45.5)	2 (22.2)	15 (40.5)	42 (62.7)	
Unknown n (%)	8 (3.7)	5 (5)	1 (11.1)	1 (2.7)	1 (1.5)	
**TPL Subtype**						*0.762**
cABO n (%)	65 (30.4)	36 (35.6)	4 (44.4)	11 (29.7)	14 (20.9)	
iABO n (%)	16 (7.5)	4 (4)	0 (0)	6 (16.2)	6 (9)	
DBD n (%)	96 (44.9)	45 (44.6)	4 (44.4)	14 (37.8)	33 (49.3)	
DBD en bloc n (%)	1 (0.5)	0 (0)	0 (0)	0 (0)	1 (1.5)	
DBD KALT n (%)	1 (0.5)	1 (1)	0 (0)	0 (0)	0 (0)	
DCD n (%)	25 (11.7)	11 (10.9)	1 (11.1)	5 (13.5)	8 (11.9)	
SLK n (%)	2 (0.9)	1 (1)	0 (0)	0 (0)	1 (1.5)	
SPK n (%)	8 (3.7)	3 (3)	0 (0)	1 (2.7)	4 (6)	
**Underlying disease**						*0.353**
Diabetic nephropathy n (%)	16 (7.5)	6 (5.9)	1 (11.1)	3 (8.1)	6 (9)	
Hypertensive nephropathy n (%)	23 (10.7)	14 (13.9)	1 (11.1)	3 (8.1)	5 (7.5)	
ADPKD n (%)	31 (14.5)	13 (12.9)	0 (0)	7 (18.9)	11 (16.4)	
GN/Vasculitis n (%)	52 (24.3)	28 (27.7)	2 (22.2)	5 (13.5)	17 (25.4)	
CAKUT n (%)	21 (9.8)	11 (10.9)	1 (11.1)	6 (16.2)	3 (4.5)	
Others n (%)	27 (12.6)	8 (7.9)	0 (0)	8 (21.6)	11 (16.4)	
Unknown n (%)	44 (20.6)	21 (20.8)	4 (44.4)	5 (13.5)	14 (20.9)	

b)	Positive Control Cohort (n=35)	TCMR IA/IB (n=9)	TCMR IIA/IIB (n=18)	Mixed histologic Rejection (n=8)	*p-values*
**Immunosuppression £**					
Tacrolimus n (%)	25 (73.5)	8 (88.9)	10 (55.6)	7 (87.5)	*0.151*
Ciclosporin n (%)	2 (5.9)	0 (0)	1 (5.6)	1 (12.5)	*0.55*
Mycophenolate n (%)	27 (79.4)	6 (66.7)	14 (77.8)	7 (87.5)	*0.521*
Azathioprin n (%)	1 (2.9)	0 (0)	0 (0)	1 (12.5)	*0.187*
Everolimus/Sirolimus n (%)	6 (17.6)	2 (22.2)	4 (22.2)	0 (0)	*0.325*
Belatacept n (%)	5 (14.7)	0 (0)	5 (27.8)	0 (0)	*0.053*
Prednisone n (%)	32 (94.1)	7 (77.8)	17 (94.4)	8 (100)	*0.052*
**Bx after TPL (y)**	1 (0-4)	1 (0-5)	0 (0-3)	5 (1-15)	*0.155*
**Presence of DSA $**					
pDSA n (%)	6 (17.1)	1 (11.1)	2 (11.1)	3 (37.5)	*0.22*
HLA Class I n (%)	2 (5.7)	0 (0)	1 (5.6)	1 (12.5)	*0.541*
HLA Class II n (%)	5 (14.3)	1 (11.1)	1 (5.6)	3 (37.5)	*0.095*
dnDSA n (%)	13 (37.1)	2 (22.2)	3 (16.7)	8 (100)	***<0.001***
HLA Class I n (%)	7 (20)	1 (11.1)	1 (5.6)	5 (62.5)	***0.003***
HLA Class II n (%)	10 (28.6)	2 (22.2)	3 (16.7)	5 (62.5)	*0.051*
**Donor age at TPL (y)**	48 (40-62)	46 (40-59)	53 (46-65)	40 (25-55)	*0.087*
**Donor sex**					*0.205**
Female n (%)	22 (62.9)	7 (77.8)	12 (66.7)	3 (37.5)	
Male (n%)	13 (37.1)	2 (22.2)	6 (33.3)	5 (62.5)	
**TPL Subtype**					*0.451**

cABO n (%)	11 (31.4)	2 (22.2)	7 (38.9)	2 (25)	
iABO n (%)	3 (8.6)	1 (11.1)	2 (11.1)	0 (0)	
DBD n (%)	16 (45.7)	5 (55.6)	6 (33.3)	5 (62.5)	
DBD en bloc n (%)	1 (2.9)	0 (0)	1 (5.6)	0 (0)	
DBD KALT n (%)	2 (5.7)	0 (0)	2 (11.1)	0 (0)	
DCD n (%)	1 (2.9)	0 (0)	0 (0)	1 (12.5)	
SLK n (%)	0 (0)	0 (0)	0 (0)	0 (0)	
SPK n (%)	1 (2.9)	1 (1)	0 (0)	0 (0)	
**Underlying disease**					*0.528**
Diabetic nephropathy n (%)	4 (11.4)	1 (11.1)	2 (11.1)	1 (12.5)	
Hypertensive nephropathy n (%)	2 (5.7)	1 (11.1)	1 (5.6)	0 (0)	
ADPKD n (%)	0 (0)	0 (0)	0 (0)	0 (0)	
GN/Vasculitis n (%)	13 (37.1)	2 (22.2)	9 (50)	2 (25)	
CAKUT n (%)	6 (17.1)	2 (22.2)	2 (11.1)	2 (25)	
Others n (%)	5 (14.3)	1 (11.1)	1 (5.6)	3 (37.5)	
Unknown n (%)	5 (14.3)	2 (22.2)	3 (16.7)	0 (0)	

Supplementary Table 1 │ Additional basic and biopsy characteristics: a) Demonstrates the Study Cohort (n=214) with its subgroups, b) demonstrates the Positive Control Cohort (n=35) with its subgroups. Continuous variables are presented as median (IQR, Q1-Q3), categorical variables as numbers (n) and percentages (of the underlying group, %). *Chi-square-test for trend. *Abbreviations: Bx, biopsy; TPL, transplantation; DSA, donor-specific antibody (p=preformed, dn=de novo); cABO, ABO-compatible; iABO, ABO-incompatible; DBD, donation after brain death; KALT, kidney after liver; DCD, donation after circulatory death; SLK, simultaneous liver-kidney; SPK, simultaneous pancreas-kidney; ADPKD, autosomal-dominant polycystic kidney disease; GN, glomerulonephritis; CAKUT, congenital anomalies of the kidney and urinary tract.*

$ One patient could have more than one DSA at a time (combinations possible).

£ One patient had no maintenance immunosuppression.

a)	**TCMR-Suspicion (n=147)**	**No Inflammation (n=67)**	***p-values***
**Female n (%)**	56 (38.1)	27 (40.3)	*0.765*
**Age at TPL (y)**	45 (34-56)	45 (36-56)	*0.639*
**Age at Bx (y)**	52 (42-61)	57 (46-64)	*0.137*
**Deceased donation n (%)**	86 (58.5)	47 (70.1)	*0.129*
**Repeat TPL n (%)**	13 (8.8)	5 (7.5)	*1*
**Bx <1y TPL n (%)**	45 (30.6)	22 (32.8)	*0.753*
**Bx indication $**			
Decrease in eGFR n (%)	77 (52.4)	28 (41.8)	*0.185*
Rise in proteinuria n (%)	40 (27.2)	24 (35.8)	*0.26*
DSA-presence n (%) $	82 (55.8)	29 (43.3)	*0.105*
**Histologic findings**			
MVI below threshold n (%) $$	21 (14.3)	17 (25.4)	*0.056*
MVI at/above threshold n (%) $$	60 (40.8)	10 (14.9)	***<0.001***
DSA- C4d- MVI n (%)	19 (12.9)	2 (3)	***0.025***
Probable hAMR n (%)	37 (25.2)	20 (29.9)	*0.507*
hAMR n (%)	45 (30.6)	8 (11.9)	***0.03***
without chronicity n (%)	12 (8.2)	2 (3)	*0.234*
DSA+, C4d- n (%)	9 (6.1)	2 (3)	*0.509*
DSA+, C4d+ n (%)	2 (1.4)	0 (0)	*1*
DSA-, C4d+ n (%)	1 (0.7)	0 (0)	*1*
cAMR n (%)	33 (22.4)	6 (9)	***0.021***
DSA+, C4d- n (%)	26 (17.7)	6 (9)	*0.059*
DSA+, C4d+ n (%)	7 (4.8)	0 (0)	*0.101*
DSA-, C4d+ n (%)	0 (0)	0 (0)	*NA*
**Immunosuppression**			
Tacrolimus n (%)	109 (74.1)	42 (62.7)	*0.106*
Ciclosporin n (%)	8 (5.4)	7 (10.4)	*0.247*
Mycophenolate n (%)	119 (81.0)	53 (79.1)	*0.853*
Azathioprin n (%)	18 (12.2)	6 (9)	*0.641*
Everolimus/Sirolimus n (%)	8 (5.4)	1 (1.5)	*0.279*
Belatacept n (%)	26 (17.7)	13 (19.4)	*0.849*
Prednisone n (%)	126 (85.7)	44 (65.7)	***0.002***
**Bx after TPL (y)**	3 (0-11)	3 (0-16)	*0.601*
**Presence of DSA $$$**			
pDSA n (%)	20 (13.6)	12 (17.9)	*0.415*
HLA Class I n (%)	11 (7.5)	4 (6)	*0.78*
HLA Class II n (%)	18 (12.2)	9 (13.4)	*1*
dnDSA n (%)	67 (45.6)	19 (28.4)	***0.024***
HLA Class I n (%)	19 (12.9)	4 (6)	*0.157*
HLA Class II n (%)	56 (38.1)	17 (25.4)	*0.087*
**Donor age at TPL (y)**	49 (42-58)	47 (39-59)	*0.634*
**Donor sex**			***0.022****
Female n (%)	77 (52.4)	24 (35.8)	
Male (n%)	63 (42.9)	42 (62.7)	
Unknown n (%)	7 (4.8)	1 (1.5)	
**TPL Subtype**			*0.344**
cABO n (%)	51 (34.7)	14 (20.9)	
iABO n (%)	10 (6.8)	6 (9)	
DBD n (%)	63 (42.9)	33 (49.3)	
DBD en bloc n (%)	0 (0)	1 (1.5)	
DBD KALT n (%)	1 (0.7)	0 (0)	
DCD n (%)	17 (11.6)	8 (11.9)	
SLK n (%)	1 (0.7)	1 (1.5)	
SPK n (%)	4 (2.7)	4 (6)	
**Underlying disease**			*0.48**
Diabetic nephropathy n (%)	10 (6.8)	6 (9)	
Hypertensive nephropathy n (%)	18 (12.2)	5 (7.5)	
ADPKD n (%)	20 (13.6)	11 (16.4)	
GN/Vasculitis n (%)	35 (23.8)	17 (25.4)	
CAKUT n (%)	18 (12.2)	3 (4.5)	
Others n (%)	16 (10.9)	11 (16.4)	
Unknown n (%)	30 (20.4)	14 (20.9)	

b)	**TCMR-Suspicion (n=147)**	**No Inflammation (n=67)**	***p-values***
**% Cortex (%)**	85 (74-91)	85 (61-90)	*0.458*
**R scores**			
R1 (no rejection)	0.74 (0.49-0.87)	0.87 (0.79-0.91)	***<0.001***
R2 (TCMR)	0.00 (0.00-0.01)	0.00 (0.00-0.00)	*0.157*

R3 (mixed)	0.00 (0.00-0.00)	0.00 (0.00-0.00)	*0.121*
R4 (early AMR)	0.06 (0.01-0.22)	0.04 (0.00-0.08)	***0.011***
R5 (full AMR)	0.02 (0.00-0.10	0.00 (0.00-0.02)	***<0.001***
R6 (late AMR)	0.04 (0.00-0.15)	0.05 (0.00-0.11)	*0.669*
R7 (all AMR)	0.23 (0.11-0.47)	0.12 (0.08-0.20)	***<0.001***
R8 (all TCMR)	0.00 (0.00-0.02)	0.00 (0.00-0.01)	***0.012***
**Mol. injury scores**			
Global disturbance	-1.13 (-2.56-0.57)	-2.55 (-3.67--1.19)	***<0.001***
AKI	0.06 (-0.39-0.48)	-0.35 (-0.64-0.05)	***<0.001***
Atrophy-Fibrosis	0.29 (0.14-0.6)	0.18 (0.1-0.43)	***0.009***
**Mol. rej. scores**			
$Rejection_{prob}$	0.08 (0.03-0.26)	0.02 (0.01-0.04)	***<0.001***
$TCMR_{prob}$	0.01 (0.01-0.02)	0.01 (0-0.01)	***0.005***
AMR_{prob}	0.06 (0.04-0.19)	0.04 (0.03-0.07)	***<0.001***
**Mol. histol. scores**			
$g>0_{prob}$	0.17 (0.1-0.38)	0.11 (0.08-0.16)	***<0.001***
$cg>0_{prob}$	0.12 (0.06-0.25)	0.08 (0.04-0.14)	***0.002***
$ptc>0_{prob}$	0.12 (0.07-0.3)	0.07 (0.05-0.1)	***<0.001***
$DSA+_{prob}$ n=114	0.46 (0.34-0.64)	0.38 (0.31-0.47)	***0.041***
$i>0_{prob}$	0.03 (0.01-0.05)	0.02 (0.01-0.02)	***<0.001***
$t>0_{prob}$	0.04 (0.02-0.06)	0.03 (0.02-0.04)	***0.001***
$ct>0_{prob}$	0.22 (0.11-0.47)	0.13 (0.08-0.31)	***0.024***
$ah>0_{prob}$ n=114	0.57 (0.44-0.68)	0.55 (0.41-0.68)	*0.692*

Supplementary Table 2 │ Basic, biopsy and MMDx characteristics between TCMR-Suspicion and No Inflammation: a) Demonstrates the basic and biopsy characteristics, b) demonstrates the MMDx characteristics. Continuous variables are presented as median (IQR, Q1-Q3), categorical variables as numbers (n) and percentages (of the underlying group, %). NA = not applicable. *Chi-square-test for trend. *Abbreviations: TPL, transplantation; Bx, biopsy; <1y, in the first year after transplantation; eGFR, estimated glomerular filtration rate; DSA, donor-specific antibody (p=preformed, dn=de novo); MVI, microvascular inflammation; (h) AMR, (histologic) antibody-mediated rejection; cAMR, chronic antibody-mediated rejection; cABO, ABO-compatible; iABO, ABO-incompatible; DBD, donation after brain death; KALT, kidney after liver; DCD, donation after circulatory death; SLK, simultaneous liver-kidney; SPK, simultaneous pancreas-kidney; ADPKD, autosomal-dominant polycystic kidney disease; GN, glomerulonephritis; CAKUT, congenital anomalies of the kidney and urinary tract; R scores, rejection phenotype scores; Mol., molecular; rej., rejection; histol., histology; prob, probability; TCMR, T cell-mediated rejection; AKI, acute kidney injury; g, glomerulitis; cg, glomerular basement membrane double contours; ptc, peritubular capillaritis; DSA, donor-specific antibody; i, interstitial inflammation; t, tubulitis; ct, tubular atrophy; ah, arteriolar hyalinosis.*

$ One patient could have more than one biopsy indication (combinations possible). DSA-presence describes the presence of any DSA (pDSA or dnDSA) with relevant mean fluorescence intensity during biopsy.
$$ All biopsies with MVI included (also if satisfying the diagnosis of AMR).
$$$ One patient could have more than one DSA at a time (combinations possible).

For explanations regarding R scores and molecular scores please refer to the manuscript.

a)	TCMR-Suspicion MVI+ (n=60)	TCMR-Suspicion MVI- (n=87)	No Inflammation MVI+ (n=10)	No Inflammation MVI- (n=57)
**Molecular rejection n (%)**	32 (53.3)	5 (5.7)	4 (40)	2 (3.5)
mAMR/TCMR n (%)	2 (3.3)	0 (0)	1 (10)	0 (0)
Histologic findings below threshold n (%)	0 (0)	0 (0)	0 (0)	0 (0)
DSA- C4d- MVI n (%)	0 (0)	0 (0)	0 (0)	0 (0)
Probable hAMR n (%)	0 (0)	0 (0)	0 (0)	0 (0)
hAMR n (%)	2 (3.3)	0 (0)	1 (10)	0 (0)
DSA+ n (%)	2 (3.3)	0 (0)	1 (10)	0 (0)
C4d+ n (%)	0 (0)	0 (0)	0 (0)	0 (0)
cg>0 n (%)	2 (3.3)	0 (0)	0 (0)	0 (0)
Minor mAMR n (%)	5 (8.3)	2 (2.3)	0 (0)	1 (1.8)
Histologic findings below threshold n (%)	0 (0)	1 (1.1)	0 (0)	0 (0)
DSA- C4d- MVI n (%)	2 (3.3)	0 (0)	0 (0)	0 (0)
Probable hAMR n (%)	0 (0)	1 (1.1)	0 (0)	1 (1.8)
hAMR n (%)	3 (5)	0 (0)	0 (0)	0 (0)
DSA+ n (%)	3 (5)	0 (0)	0 (0)	0 (0)
C4d+ n (%)	2 (3.3)	0 (0)	0 (0)	0 (0)
cg>0 n (%)	3 (5)	0 (0)	0 (0)	0 (0)
mAMR n (%)	25 (41.7)	3 (3.4)	3 (30)	1 (1.8)
Histologic findings below threshold n (%)	0 (0)	1 (1.1)	0 (0)	1 (1.8)
DSA- C4d- MVI n (%)	8 (13.3)	0 (0)	0 (0)	0 (0)
Probable hAMR n (%)	0 (0)	1 (1.1)	0 (0)	0 (0)
hAMR n (%)	17 (28.3)	1 (1.1)	3 (30)	0 (0)
DSA+ n (%)	17 (28.3)	1 (1.1)	3 (30)	0 (0)
C4d+ n (%)	1 (1.7)	1 (1.1)	0 (0)	0 (0)
cg>0 n (%)	13 (21.7)	1 (1.1)	2 (20)	0 (0)
**No molecular rejection n (%)**	28 (46.7)	82 (94.3)	6 (60)	55 (96.5)
Histologic findings below threshold n (%)	0 (0)	44 (50.6)	0 (0)	36 (63.2)
DSA- C4d- MVI n (%)	9 (15)	0 (0)	2 (20)	0 (0)
Probable hAMR n (%)	0 (0)	35 (40.2)	0 (0)	19 (33.3)
hAMR n (%)	19 (31.7)	3 (3.4)	4 (40)	0 (0)
DSA+ n (%)	19 (31.7)	2 (2.3)	4 (40)	0 (0)
C4d+ n (%)	3 (5)	3 (3.4)	0 (0)	0 (0)
cg>0 n (%)	14 (23.3)	0 (0)	4 (40)	0 (0)

b)	Isolated Tubulitis MVI+ (n=36)	Isolated Tubulitis MVI- (n=65)	Border-line Changes MVI+ (n=5)	Border-line Changes MVI- (n=4)	Isolated Arteritis MVI+ (n=19)	Isolated Arteritis MVI -(n=18)
**Molecular rejection n (%)**	19 (52.8)	4 (6.2)	4 (80)	1 (25)	9 (47.4)	0 (0)
mAMR/TCMR n (%)	1 (2.8)	0 (0)	1 (20)	0 (0)	0 (0)	0 (0)
Histologic findings below threshold n (%)	0 (0)	0 (0)	0 (0)	0 (0)	0 (0)	0 (0)
DSA- C4d- MVI n (%)	0 (0)	0 (0)	0 (0)	0 (0)	0 (0)	0 (0)
Probable hAMR n (%)	0 (0)	0 (0)	0 (0)	0 (0)	0 (0)	0 (0)
hAMR n (%)	1 (2.8)	0 (0)	1 (20)	0 (0)	0 (0)	0 (0)
DSA+ n (%)	1 (2.8)	0 (0)	1 (20)	0 (0)	0 (0)	0 (0)
C4d+ n (%)	0 (0)	0 (0)	0 (0)	0 (0)	0 (0)	0 (0)
cg>0 n (%)	1 (2.8)	0 (0)	1 (20)	0 (0)	0 (0)	0 (0)
Minor mAMR n (%)	4 (11.1)	2 (3.1)	0 (0)	0 (0)	1 (5.3)	0 (0)
Histologic findings below threshold n (%)	0 (0)	1 (1.5)	0 (0)	0 (0)	0 (0)	0 (0)
DSA- C4d- MVI n (%)	2 (5.6)	0 (0)	0 (0)	0 (0)	0 (0)	0 (0)
Probable hAMR n (%)	0 (0)	1 (1.5)	0 (0)	0 (0)	0 (0)	0 (0)
hAMR n (%)	2 (5.6)	0 (0)	0 (0)	0 (0)	1 (5.3)	0 (0)
DSA+ n (%)	2 (5.6)	0 (0)	0 (0)	0 (0)	1 (5.3)	0 (0)
C4d+ n (%)	1 (2.8)	0 (0)	0 (0)	0 (0)	1 (5.3)	0 (0)
cg>0 n (%)	2 (5.6)	0 (0)	0 (0)	0 (0)	1 (5.3)	0 (0)
mAMR n (%)	14 (38.9)	2 (3.1)	3 (60)	1 (25)	8 (42.1)	0 (0)
Histologic findings below threshold n (%)	0 (0)	1 (1.5)	0 (0)	0 (0)	0 (0)	0 (0)
DSA- C4d- MVI n (%)	3 (8.3)	0 (0)	1 (20)	0 (0)	4 (21.1)	0 (0)
Probable hAMR n (%)	0 (0)	0 (0)	0 (0)	1 (25)	0 (0)	0 (0)
hAMR n (%)	11 (30.6)	1 (1.5)	2 (40)	0 (0)	4 (21.1)	0 (0)
DSA+ n (%)	11 (30.6)	1 (1.5)	2 (40)	0 (0)	4 (21.1)	0 (0)
C4d+ n (%)	1 (2.8)	1 (1.5)	0 (0)	0 (0)	0 (0)	0 (0)
cg>0 n (%)	10 (27.8)	1 (1.5)	1 (20)	0 (0)	2 (10.5)	0 (0)

**No molecular rejection n (%)**	17 (47.2)	61 (93.8)	1 (20)	3 (75)	10 (52.6)	18 (100)
Histologic findings below threshold n (%)	0 (0)	32 (49.2)	0 (0)	2 (50)	0 (0)	10 (55.6)
DSA- C4d- MVI n (%)	3 (8.3)	0 (0)	1 (20)	0 (0)	5 (26.3)	0 (0)
Probable hAMR n (%)	0 (0)	27 (41.5)	0 (0)	1 (25)	0 (0)	7 (38.9)
hAMR n (%)	14 (38.9)	2 (3.1)	0 (0)	0 (0)	5 (26.3)	1 (5.6)
DSA+ n (%)	14 (38.9)	2 (3.1)	0 (0)	0 (0)	5 (26.3)	0 (0)
C4d+ n (%)	2 (5.6)	2 (3.1)	0 (0)	0 (0)	1 (5.3)	1 (5.6)
cg>0 n (%)	10 (27.8)	0 (0)	0 (0)	0 (0)	5 (26.3)	0 (0)

c)	**Positive Control Cohort MVI+ (n=22)**	**Positive Control Cohort MVI- (n=13)**
**Molecular rejection n (%)**	19 (86.4)	11 (84.6)
mTCMR n (%)	7 (31.8)	3 (23.1)
Histologic findings below threshold n (%)	0 (0)	3 (23.1)
DSA- C4d- MVI n (%)	6 (27.3)	0 (0)
Probable hAMR n (%)	0 (0)	0 (0)
hAMR n (%)	1 (4.5)	0 (0)
DSA+ n (%)	1 (4.5)	0 (0)
C4d+ n (%)	0 (0)	0 (0)
cg>0 n (%)	0 (0)	0 (0)
mAMR/TCMR n (%)	5 (22.7)	7 (53.8)
Histologic findings below threshold n (%)	0 (0)	4 (30.8)
DSA- C4d- MVI n (%)	1 (4.5)	0 (0)
Probable hAMR n (%)	0 (0)	3 (23.1)
hAMR n (%)	4 (18.2)	0 (0)
DSA+ n (%)	4 (18.2)	0 (0)
C4d+ n (%)	1 (4.5)	0 (0)
cg>0 n (%)	0 (0)	0 (0)
Minor mAMR n (%)	0 (0)	1 (7.7)
Histologic findings below threshold n (%)	0 (0)	0 (0)
DSA- C4d- MVI n (%)	0 (0)	0 (0)
Probable hAMR n (%)	0 (0)	1 (7.7)
hAMR n (%)	0 (0)	0 (0)
DSA+ n (%)	0 (0)	0 (0)
C4d+ n (%)	0 (0)	0 (0)
cg>0 n (%)	0 (0)	0 (0)
mAMR n (%)	7 (31.8)	0 (0)
Histologic findings below threshold n (%)	0 (0)	0 (0)
DSA- C4d- MVI n (%)	5 (22.7)	0 (0)
Probable hAMR n (%)	0 (0)	0 (0)
hAMR n (%)	2 (9.1)	0 (0)
DSA+ n (%)	2 (9.1)	0 (0)
C4d+ n (%)	1 (4.5)	0 (0)
cg>0 n (%)	2 (9.1)	0 (0)
**No molecular rejection n (%)**	3 (13.6)	2 (15.4)
Histologic findings below threshold n (%)	0 (0)	2 (15.4)
DSA- C4d- MVI n (%)	2 (9.1)	0 (0)
Probable hAMR n (%)	0 (0)	0 (0)
hAMR n (%)	1 (4.5)	0 (0)
DSA+ n (%)	1 (4.5)	0 (0)
C4d+ n (%)	1 (4.5)	0 (0)
cg>0 n (%)	0 (0)	0 (0)

d)	**TCMR IA/IIB (n=9)**	**TCMR IIA/IIB (n=18)**	**Mixed histologic Rejection (n=8)**
**Molecular rejection n (%)**	9 (100)	14 (77.8)	7 (87.5)
mTCMR n (%)	4 (44.4)	5 (27.8)	1 (12.5)
Histologic findings below threshold n (%)	1 (11.1)	2 (11.1)	0 (0)
DSA- C4d- MVI n (%)	3 (33.3)	3 (16.7)	0 (0)
Probable hAMR n (%)	0 (0)	0 (0)	0 (0)
hAMR n (%)	0 (0)	0 (0)	1 (12.5)
DSA+ n (%)	0 (0)	0 (0)	1 (12.5)
C4d+ n (%)	0 (0)	0 (0)	0 (0)
cg>0 n (%)	0 (0)	0 (0)	0 (0)
mAMR/TCMR n (%)	4 (44.4)	4 (22.2)	4 (50)

Histologic findings below threshold n (%)	2 (22.2)	2 (11.1)	0 (0)
DSA- C4d- MVI n (%)	1 (11.1)	0 (0)	0 (0)
Probable hAMR n (%)	1 (11.1)	2 (11.1)	0 (0)
hAMR n (%)	0 (0)	0 (0)	4 (50)
DSA+ n (%)	0 (0)	0 (0)	4 (50)
C4d+ n (%)	0 (0)	0 (0)	1 (12.5)
cg>0 n (%)	0 (0)	0 (0)	0 (0)
Minor mAMR n (%)	0 (0)	1 (5.6)	0 (0)
Histologic findings below threshold n (%)	0 (0)	0 (0)	0 (0)
DSA- C4d- MVI n (%)	0 (0)	0 (0)	0 (0)
Probable hAMR n (%)	0 (0)	1 (5.6)	0 (0)
hAMR n (%)	0 (0)	0 (0)	0 (0)
DSA+ n (%)	0 (0)	0 (0)	0 (0)
C4d+ n (%)	0 (0)	0 (0)	0 (0)
cg>0 n (%)	0 (0)	0 (0)	0 (0)
mAMR n (%)	1 (11.1)	4 (22.2)	2 (25)
Histologic findings below threshold n (%)	0 (0)	0 (0)	0 (0)
DSA- C4d- MVI n (%)	1 (11.1)	4 (22.2)	0 (0)
Probable hAMR n (%)	0 (0)	0 (0)	0 (0)
hAMR n (%)	0 (0)	0 (0)	2 (25)
DSA+ n (%)	0 (0)	0 (0)	2 (25)
C4d+ n (%)	0 (0)	0 (0)	1 (12.5)
cg>0 n (%)	0 (0)	0 (0)	2 (25)
**No molecular rejection n (%)**	0 (0)	4 (22.2)	1 (12.5)
Histologic findings below threshold n (%)	0 (0)	2 (11.1)	0 (0)
DSA- C4d- MVI n (%)	0 (0)	2 (11.1)	0 (0)
Probable hAMR n (%)	0 (0)	0 (0)	0 (0)
hAMR n (%)	0 (0)	0 (0)	1 (12.5)
DSA+ n (%)	0 (0)	0 (0)	1 (12.5)
C4d+ n (%)	0 (0)	0 (0)	1 (12.5)
cg>0 n (%)	0 (0)	0 (0)	0 (0)

Supplementary Table 3 │ Histology details of molecular phenotypes in subgroups: a) Demonstrates TCMR-Suspicion and No Inflammation, b) demonstrates the subgroups of TCMR-Suspicion and c) demonstrates the Positive Control Cohort. All histology details a-c are presented based on the presence/absence of MVI at/above threshold. d) presents the Positive Control Cohort subdivided into the subgroups from the main manuscript (TCMR IA/IB, TCMR IIA/IIB, Mixed histologic Rejection). Categorical variables as numbers (n) and percentages (of the underlying group, %). The prefix «m» in front of every rejection phenotype stands for «molecular». *Abbreviations: TCMR, t cell-mediated rejection; AMR, antibody-mediated rejection, DSA, donor-specific antibody.*

a)	Study Cohort (n=214)	Isolated Tubulitis (n=101)	Borderline Changes (n=9)	Isolated Arteritis (n=37)	No Inflammation (n=67)
**Molecular rejection with hAMR n (%)**	27 (12.6)	15 (14.9)	3 (33.3)	5 (13.5)	4 (6)
mTCMR n (%)	0 (0)	0 (0)	0 (0)	0 (0)	0 (0)
mAMR/TCMR n (%)	3 (1.4)	1 (1)	1 (11.1)	0 (0)	1 (1.4)
Minor mAMR n (%)	3 (1.4)	2 (2)	0 (0)	1 (2.7)	0 (0)
mAMR n (%)	21 (9.8)	12 (11.9)	2 (22.2)	4 (10.8)	3 (4.5)
**Molecular rejection without hAMR n (%)**	16 (7.5)	8 (7.9)	2 (22.2)	4 (10.8)	2 (3)
mTCMR n (%)	0 (0)	0 (0)	0 (0)	0 (0)	0 (0)
mAMR/TCMR n (%)	0 (0)	0 (0)	0 (0)	0 (0)	0 (0)
Minor mAMR n (%)	5 (2.3)	4 (4)	0 (0)	0 (0)	1 (1.4)
mAMR n (%)	11 (5.1)	4 (4)	2 (22.2)	4 (10.8)	1 (1.4)
**No molecular rejection with hAMR n (%)**	26 (12.1)	16 (15.8)	0 (0)	6 (16.2)	4 (6)
**No molecular rejection without hAMR n (%)**	145 (67.8)	62 (61.4)	4 (44.4)	22 (59.5)	57 (85.1)

b)	Positive Control Cohort (n=35)	TCMR IA/IB (n=9)	TCMR IIA/IIB (n=18)	Mixed histologic Rejection (n=8)
**Molecular rejection with hAMR n (%)**	7 (20)	0 (0)	0 (0)	7 (87.5)
mTCMR n (%)	1 (2.8)	0 (0)	0 (0)	1 (12.5)
mAMR/TCMR n (%)	4 (11.4)	0 (0)	0 (0)	4 (50)
Minor mAMR n (%)	0 (0)	0 (0)	0 (0)	0 (0)
mAMR n (%)	2 (5.7)	0 (0)	0 (0)	2 (25)
**Molecular rejection without hAMR n (%)**	23 (65.7)	9 (100)	14 (77.8)	0 (0)
mTCMR n (%)	9 (25.7)	4 (44.4)	5 (27.8)	0 (0)
mAMR/TCMR n (%)	8 (22.9)	4 (44.4)	4 (22.2)	0 (0)
Minor mAMR n (%)	1 (2.8)	0 (0)	1 (5.6)	0 (0)
mAMR n (%)	5 (14.3)	1 (11.1)	4 (22.2)	0 (0)
**No molecular rejection with hAMR n (%)**	1 (2.9)	0 (0)	0 (0)	1 (12.5)
**No molecular rejection without hAMR n (%)**	4 (11.4)	0 (0)	4 (22.2)	0 (0)

c)	*Comment on Suppl. Table 3a*
**TCMR-Suspicion with MVI**	**22 of 32 (68.9%)** molecular rejections had hAMR
**TCMR-Suspicion without MVI**	1 of 5 (20%) molecular rejections had hAMR
**No Inflammation with MVI**	**4 of 4 (100%)** molecular rejections had hAMR
**No Inflammation without MVI**	0 of 2 (0%) molecular rejections had hAMR

Supplementary Table 4 │ Correlation of molecular rejections with hAMR among subgroups: a) Demonstrates the Study Cohort (n=214) and b) demonstrates the Positive Control Cohort (n=35) regarding the number (n) of molecular rejections depending on hAMR. c) Demonstrates a summary oft he number (n) of molecular rejections with hAMR subdivided based on TCMR-Suspicion with/without MVI and No Inflammation with/without MVI. Notably, only 22/32 (68.9%) of molecular rejections (either mAMR/TCMR, minor mAMR or mAMR) in the group of TCMR-Suspicion with MVI fulfilled histologic criteria for AMR (hAMR), whereas 10/32 (31.1%) of molecular rejections in this subgroup did not fulfill hAMR-criteria. *Abbreviations: hAMR, histologic antibody-mediated rejection, TCMR, t cell-mediated rejection; MVI, microvascular inflammation.*

	TCMR1-phenotype (n=18)	TCMR2-phenotype (n=17)	*p-values*
**Histologic Characteristics**			
AMR-related lesions			
Findings below threshold n (%)	3 (16.7)	6 (35.3)	*0.264*
Probable hAMR n (%)	4 (22.2)	0 (0.0)	*0.104*
DSA- C4d- MVI n (%)	6 (33.3)	8 (47.1)	*0.5*
hAMR n (%)	5 (27.8)	3 (17.6)	*0.691*
TCMR-related lesions			
TCMR IA/IB n (%)	3 (16.7)	6 (35.3)	*0.264*
TCMR IIA/IIB n (%)	10 (55.6)	8 (47.1)	*0.74*
Mixed histologic Rejection n (%)	5 (27.8)	3 (17.6)	*0.691*
caTCMR n (%)	5 (27.8)	3 (17.6)	*0.691*
**Chronicity lesions**			
ci>1 n (%)*	4 (22.2)	4 (23.5)	*1*
ct>1 n (%)	4 (22.2)	4 (23.5)	*1*
cv>1 n (%)*	7 (38.9)	2 (11.8)	*0.125*
cg>1 n (%)*	4 (22.2)	1 (5.9)	*0.335*
ci+ct+cv+2xcg>9 n (%)	3 (16.7)	1 (5.9)	*0.603*
**Molecular phenotypes**			
mTCMR n (%)	0 (0)	10 (58.8)	***<0.001***
mAMR/TCMR n (%)	7 (38.9)	5 (29.4)	*0.725*
Minor mAMR n (%)	1 (5.6)	0 (0.0)	*1*
mAMR n (%)	6 (33.3)	1 (5.9)	*0.088*
No molecular rejection n (%)	4 (22.2)	1 (5.9)	*0.338*

Supplementary Table 5 │ Subgroups of TCMR1 and TCMR2 among the Positive Control Cohort: All cases of the Positive Control Cohort (n=35) were categorized into either TCMR1 (formerly mixed) or TCMR2 based on their archetypal analysis (either R2<R3 or R2>R3). Fisher's exact test was used for comparison. *Abbreviations: AMR, antibody-mediated rejection, DSA, donor-specific antibody; MVI, microvascular inflammation; TCMR, t cell-mediated rejection (ca=chronic-active); ci, interstitial fibrosis; ct, tubular atrophy; cv, vascular fibrous intimal thickening; cg, glomerular basement membrane double contours.*

a)	Study Cohort (n=214)	Isolated Tubulitis (n=101)	Borderline Changes (n=9)	Isolated Arteritis (n=37)	No Inflammation (n=67)	*p-values*
i						***<0.001****
0 n (%)	204 (95.3)	101 (100)	0 (0)	36 (97.3)	67 (100)	
1 n (%)	10 (4.7)	0 (0)	9 (100)	1 (2.7)	0 (0)	
2 n (%)	0 (0)	0 (0)	0 (0)	0 (0)	0 (0)	
3 n (%)	0 (0)	0 (0)	0 (0)	0 (0)	0 (0)	
t						***<0.001****
0 n (%)	78 (36.4)	0 (0)	0 (0)	11 (29.7)	67 (100)	
1 n (%)	121 (56.5)	87 (86.1)	9 (100)	25 (67.6)	0 (0)	
2 n (%)	14 (6.5)	13 (12.9)	0 (0)	1 (2.7)	0 (0)	
3 n (%)	1 (0.5)	1 (1)	0 (0)	0 (0)	0 (0)	
v						***<0.001****
0 n (%)	177 (82.7)	101 (100)	9 (100)	0 (0)	67 (100)	
1 n (%)	37 (17.3)	0 (0)	0 (0)	37 (100)	0 (0)	
2 n (%)	0 (0)	0 (0)	0 (0)	0 (0)	0 (0)	
3 n (%)	0 (0)	0 (0)	0 (0)	0 (0)	0 (0)	
g						***<0.001****
0 n (%)	109 (50.9)	55 (54.5)	3 (33.3)	11 (29.7)	40 (59.7)	
1 n (%)	47 (22)	16 (15.8)	4 (44.4)	9 (24.3)	18 (26.9)	
2 n (%)	48 (22.4)	21 (20.8)	2 (22.2)	16 (43.2)	9 (13.4)	
3 n (%)	10 (4.7)	9 (8.9)	0 (0)	1 (2.7)	0 (0)	
ptc						***<0.001****
0 n (%)	167 (78)	76 (75.2)	3 (33.3)	25 (67.6)	63 (94)	
1 n (%)	10 (4.7)	5 (5)	3 (33.3)	2 (5.4)	0 (0)	
2 n (%)	31 (14.5)	18 (17.8)	3 (33.3)	8 (21.6)	2 (3)	
3 n (%)	6 (2.8)	2 (2)	0 (0)	2 (5.4)	2 (3)	
ti						***<0.001****
0 n (%)	153 (71.5)	68 (67.3)	1 (11.1)	28 (75.7)	56 (83.6)	
1 n (%)	51 (23.8)	27 (26.7)	6 (66.7)	8 (21.6)	10 (14.9)	
2 n (%)	8 (3.7)	6 (5.9)	2 (22.2)	0 (0)	0 (0)	
3 n (%)	2 (0.9)	0 (0)	0 (0)	1 (2.7)	1 (1.5)	
i-IFTA						***<0.001****
0 n (%)	101 (47.2)	36 (35.6)	1 (11.1)	20 (54.1)	44 (65.7)	
1 n (%)	30 (14)	14 (13.9)	4 (44.4)	4 (10.8)	8 (11.9)	
2 n (%)	39 (18.2)	25 (24.8)	1 (11.1)	4 (10.8)	9 (13.4)	
3 n (%)	44 (20.6)	26 (25.7)	3 (33.3)	9 (24.3)	6 (9)	
t-IFTA n=142						***0.003****
0 n (%)	79 (36.9)	32 (31.7)	1 (11.1)	12 (32.4)	34 (50.7)	
1 n (%)	50 (23.4)	36 (35.6)	0 (0)	7 (18.9)	7 (10.4)	
2 n (%)	11 (5.1)	3 (3)	1 (11.1)	4 (10.8)	3 (4.5)	
3 n (%)	2 (0.9)	2 (2)	0 (0)	0 (0)	0 (0)	
Unknown n (%)	72 (33.6)	28 (27.7)	7 (77.8)	14 (37.8)	23 (34.3)	
C4d						*0.062**
0 n (%)	164 (76.6)	73 (72.3)	6 (66.7)	30 (81.1)	55 (82.1)	
1 n (%)	24 (11.2)	16 (15.8)	3 (33.3)	1 (2.7)	4 (6)	
2 n (%)	6 (2.8)	2 (2)	0 (0)	3 (8.1)	1 (1.5)	
3 n (%)	20 (9.3)	10 (9.9)	0 (0)	3 (8.1)	7 (10.4)	
cg n=212						***<0.001****
0 n (%)	108 (50.5)	44 (43.6)	3 (33.3)	25 (67.6)	36 (53.7)	
1 n (%) $	70 (32.7)	37 (36.6)	3 (33.3)	5 (13.5)	25 (37.3)	
2 n (%)	7 (3.3)	3 (3)	2 (22.2)	1 (2.7)	1 (1.5)	
3 n (%)	27 (12.6)	16 (15.8)	0 (0)	6 (16.2)	5 (7.5)	
Unknown n (%)	2 (0.9)	1 (1)	1 (11.1)	0 (0)	0 (0)	
mm n=208						*0.901**
0 n (%)	185 (86.4)	86 (85.1)	8 (88.9)	35 (94.6)	56 (83.6)	
1 n (%)	14 (6.5)	5 (5)	1 (11.1)	2 (5.4)	6 (9)	
2 n (%)	2 (0.9)	1 (1)	0 (0)	0 (0)	1 (1.5)	
3 n (%)	7 (3.3)	4 (4)	0 (0)	0 (0)	3 (4.5)	
Unknown n (%)	6 (2.8)	5 (5)	0 (0)	0 (0)	1 (1.5)	
ci						*0.289**
0 n (%)	54 (25.2)	22 (21.8)	1 (11.1)	8 (21.6)	23 (34.3)	
1 n (%)	124 (57.9)	64 (63.4)	5 (55.6)	23 (62.2)	32 (47.8)	
2 n (%)	25 (11.7)	13 (12.9)	2 (22.2)	3 (8.1)	7 (10.4)	
3 n (%)	11 (5.1)	2 (2)	1 (11.1)	3 (8.1)	5 (7.5)	
ct						*0.374**
0 n (%)	11 (5.1)	3 (3)	0 (0)	4 (10.8)	4 (6)	

1 n (%)	167 (78)	83 (82.2)	6 (66.7)	27 (73)	51 (76.1)	
2 n (%)	25 (11.7)	13 (12.9)	2 (22.2)	3 (8.1)	7 (10.4)	
3 n (%)	11 (5.1)	2 (2)	1 (11.1)	3 (8.1)	5 (7.5)	
cv						*0.093**
0 n (%)	35 (16.4)	18 (17.8)	4 (44.4)	3 (8.1)	10 (14.9)	
1 n (%)	106 (49.5)	53 (52.5)	3 (33.3)	14 (37.8)	36 (53.7)	
2 n (%)	48 (22.4)	19 (18.8)	2 (22.2)	12 (32.4)	15 (22.4)	
3 n (%)	25 (11.7)	11 (10.9)	0 (0)	8 (21.6)	6 (9)	
ah						*0.664**
0 n (%)	27 (12.6)	10 (9.9)	1 (11.1)	6 (16.2)	10 (14.9)	
1 n (%)	74 (34.6)	30 (29.7)	5 (55.6)	13 (35.1)	26 (38.8)	
2 n (%)	70 (32.7)	39 (38.6)	2 (22.2)	12 (32.4)	17 (25.4)	
3 n (%)	43 (20.1)	22 (21.8)	1 (11.1)	6 (16.2)	14 (20.9)	
aah						***0.021****
0 n (%)	104 (48.6)	38 (37.6)	7 (77.8)	19 (51.4)	40 (59.7)	
1 n (%)	40 (18.7)	21 (20.8)	1 (11.1)	10 (27.0)	8 (11.9)	
2 n (%)	70 (32.7)	42 (41.6)	1 (11.1)	8 (21.6)	19 (28.4)	
3 n (%)	0 (0)	0 (0)	0 (0)	0 (0)	0 (0)	
ptcml n=120						*0.225**
0 n (%)	113 (52.8)	59 (58.4)	2 (22.2)	16 (43.2)	36 (53.7)	
1 n (%)	7 (3.3)	4 (4)	0 (0)	2 (5.4)	1 (1.5)	
Unknown n (%)	94 (43.9)	38 (37.6)	7 (77.8)	19 (51.4)	30 (44.8)	

b)	**Positive Control Cohort (n=35)**	**TCMR IA/IB (n=9)**	**TCMR IIA/IIB (n=18)**	**Mixed histologic Rejection (n=8)**	***p-values***
i					*0.262**
0 n (%)	0 (0)	0 (0)	0 (0)	0 (0)	
1 n (%)	12 (34.3)	1 (11.1)	6 (33.3)	5 (62.5)	
2 n (%)	13 (37.1)	4 (44.4)	7 (38.9)	2 (25)	
3 n (%)	10 (28.6)	4 (44.4)	5 (27.8)	1 (12.5)	
t					*0.267**
0 n (%)	0 (0)	0 (0)	0 (0)	0 (0)	
1 n (%)	10 (28.6)	0 (0)	7 (38.9)	3 (37.5)	
2 n (%)	10 (28.6)	3 (33.3)	5 (27.8)	2 (25)	
3 n (%)	15 (42.9)	6 (66.7)	6 (33.3)	3 (37.5)	
v n=34					***<0.001****
0 n (%)	10 (28.6)	9 (100)	0 (0)	1 (12.5)	
1 n (%)	21 (60)	0 (0)	16 (88.9)	5 (62.5)	
2 n (%)	3 (8.6)	0 (0)	2 (11.1)	1 (12.5)	
3 n (%)	0 (0)	0 (0)	0 (0)	0 (0)	
Unknown n (%)	1 (2.9)	0 (0)	0 (0)	1 (12.5)	
g n=34					*0.355**
0 n (%)	9 (25.7)	4 (44.4)	5 (27.8)	0 (0)	
1 n (%)	15 (42.9)	2 (22.2)	8 (44.4)	5 (62.5)	
2 n (%)	8 (22.9)	3 (33.3)	3 (16.7)	2 (25)	
3 n (%)	2 (5.7)	0 (0)	1 (5.6)	1 (12.5)	
Unknown n (%)	1 (2.9)	0 (0)	1 (5.6)	0 (0)	
ptc					*0.761**
0 n (%)	9 (25.7)	3 (33.3)	5 (27.8)	1 (12.5)	
1 n (%)	5 (14.3)	1 (11.1)	2 (11.1)	2 (25)	
2 n (%)	17 (48.6)	4 (44.4)	8 (44.4)	5 (62.5)	
3 n (%)	4 (11.4)	1 (11.1)	3 (16.7)	0 (0)	
ti					*0.376**
0 n (%)	2 (5.7)	1 (11.1)	1 (5.6)	0 (0)	
1 n (%)	9 (25.7)	0 (0)	5 (27.8)	4 (50)	
2 n (%)	13 (37.1)	5 (55.6)	6 (33.3)	2 (25)	
3 n (%)	11 (31.4)	3 (33.3)	6 (33.3)	2 (25)	
i-IFTA					*0.439**
0 n (%)	7 (20)	1 (11.1)	4 (22.2)	2 (25)	
1 n (%)	4 (11.4)	0 (0)	2 (11.1)	2 (25)	
2 n (%)	3 (8.6)	0 (0)	2 (11.1)	1 (12.5)	
3 n (%)	21 (60)	8 (88.9)	10 (55.6)	3 (37.5)	
t-IFTA n=14					*0.069**
0 n (%)	6 (17.1)	0 (0)	4 (22.2)	2 (25)	
1 n (%)	2 (5.7)	0 (0)	0 (0)	2 (25)	
2 n (%)	6 (17.1)	3 (33.3)	2 (11.1)	1 (12.5)	
3 n (%)	0 (0)	0 (0)	0 (0)	0 (0)	

Unknown n (%)	21 (60)	6 (66.7)	12 (66.7)	3 (37.5)	
C4d					*0.158**
0 n (%)	26 (74.3)	8 (88.9)	15 (83.3)	3 (37.5)	
1 n (%)	3 (8.6)	0 (0)	1 (5.6)	2 (25)	
2 n (%)	3 (8.6)	0 (0)	1 (5.6)	2 (25)	
3 n (%)	3 (8.6)	1 (11.1)	1 (5.6)	1 (12.5)	
cg n=34					*0.700**
0 n (%)	22 (62.9)	6 (66.7)	10 (55.6)	6 (75)	
1 n (%) $	7 (20)	3 (33.3)	3 (16.7)	1 (12.5)	
2 n (%)	1 (2.9)	0 (0)	1 (5.6)	0 (0)	
3 n (%)	4 (11.4)	0 (0)	3 (16.7)	1 (12.5)	
Unknown n (%)	1 (2.9)	0 (0)	1 (5.6)	0 (0)	
mm n=32					*NA*
0 n (%)	32 (91.4)	8 (88.9)	17 (94.4)	7 (87.5)	
1 n (%)	0 (0)	0 (0)	0 (0)	0 (0)	
2 n (%)	0 (0)	0 (0)	0 (0)	0 (0)	
3 n (%)	0 (0)	0 (0)	0 (0)	0 (0)	
Unknown n (%)	3 (8.6)	1 (11.1)	1 (5.6)	1 (12.5)	
ci n=34					*0.694**
0 n (%)	5 (14.3)	2 (22.2)	2 (11.1)	1 (12.5)	
1 n (%)	21 (60)	5 (55.6)	11 (61.1)	5 (62.5)	
2 n (%)	7 (20)	1 (11.1)	4 (22.2)	2 (25)	
3 n (%)	1 (2.9)	1 (11.1)	0 (0)	0 (0)	
Unknown n (%)	1 (2.9)	0 (0)	1 (5.6)	0 (0)	
ct					*0.694**
0 n (%)	3 (8.6)	1 (11.1)	1 (5.6)	1 (12.5)	
1 n (%)	24 (68.6)	6 (66.7)	13 (72.2)	5 (62.5)	
2 n (%)	7 (20)	1 (11.1)	4 (22.2)	2 (25)	
3 n (%)	1 (2.9)	1 (11.1)	0 (0)	0 (0)	
cv n=34					*0.726**
0 n (%)	8 (22.9)	3 (33.3)	4 (22.2)	1 (12.5)	
1 n (%)	17 (48.6)	5 (55.6)	9 (50)	3 (37.5)	
2 n (%)	5 (14.3)	1 (11.1)	3 (16.7)	1 (12.5)	
3 n (%)	4 (11.4)	0 (0)	2 (11.1)	2 (25)	
Unknown n (%)	1 (2.9)	0 (0)	0 (0)	1 (12.5)	
ah					*0.745**
0 n (%)	8 (22.9)	1 (11.1)	5 (27.8)	2 (25)	
1 n (%)	16 (45.7)	5 (55.6)	7 (38.9)	4 (50)	
2 n (%)	4 (11.4)	2 (22.2)	2 (11.1)	0 (0)	
3 n (%)	7 (20)	1 (11.1)	4 (22.2)	2 (25)	
aah					*0.919**
0 n (%)	23 (65.7)	7 (77.8)	11 (61.1)	5 (62.5)	
1 n (%)	4 (11.4)	1 (11.1)	2 (11.1)	1 (12.5)	
2 n (%)	6 (17.1)	1 (11.1)	4 (22.2)	1 (12.5)	
3 n (%)	2 (5.7)	0 (0)	1 (5.6)	1 (12.5)	
ptcml n=10					*0.732**
0 n (%)	8 (22.9)	2 (22.2)	3 (16.7)	3 (37.5)	
1 n (%)	2 (5.7)	0 (0)	1 (5.6)	1 (12.5)	
Unknown n (%)	25 (71.4)	7 (77.8)	14 (77.8)	4 (50)	

Supplementary Table 6 │ Detailed Banff-lesions of all subgroups: a) Demonstrates the Study Cohort (n=214), b) demonstrates the Positive Control Cohort (n=35). Categorical variables as numbers (n) and percentages (of the underlying group, %). *Chi-square test for trend. *Abbreviations: i, interstitial inflammation; t, tubulitis; v, intimal arteritis; g, glomerulitis; ptc, peritubular capillaritis; ti, total inflammation; i-IFTA, inflammation in areas of IFTA (interstitial fibrosis and tubular atrophy); t-IFTA, tubulitis in areas of IFTA; cg, glomerular basement membrane double contours; mm, mesangial matrix expansion; ci, interstitial fibrosis; ct, tubular atrophy; cv, vascular fibrous intimal thickening; ah, arteriolar hyalinosis; aah, hyaline arteriolar thickening; ptcml, peritubular capillary basement membrane multilayering.*

a)	Protocol (n=21)	Only eGFR (n=56)	Only Proteinuria (n=23)	Only DSA (n=56)	eGFR + DSA (n=42)	eGFR + Proteinuria (n=22)	Proteinuria + DSA (n=15)	eGFR + Proteinuria + DSA (n=14)
**Molecular rejection n (%)**	3 (14.3)	15 (26.8)	4 (17.4)	8 (14.3)	22 (52.4)	7 (31.8)	8 (53.3)	6 (42.9)
mTCMR n (%)	0 (0)	7 (12.5)	0 (0)	0 (0)	3 (7.1)	0 (0)	0 (0)	0 (0)
mAMR/TCMR n (%)	0 (0)	3 (5.4)	0 (0)	3 (5.4)	7 (16.7)	0 (0)	1 (6.7)	1 (6.7)
Minor mAMR n (%)	1 (4.8)	1 (1.8)	1 (4.3)	1 (1.8)	2 (4.8)	1 (4.5)	1 (6.7)	1 (6.7)
mAMR n (%)	2 (9.5)	4 (7.1)	3 (13)	4 (7.1)	10 (23.8)	6 (27.3)	6 (40)	4 (28.6)
**No molecular rejection n (%)**	18 (85.7)	41 (73.2)	19 (82.6)	48 (85.7)	20 (47.6)	15 (68.2)	7 (46.7)	8 (57.1)

b)	Protocol (n=21)	For Cause (n=228)
**Molecular rejection n (%)**	3 (14.3)	70 (30.7)
mTCMR n (%)	0 (0)	10 (4.4)
mAMR/TCMR n (%)	0 (0)	15 (6.6)
Minor mAMR n (%)	1 (4.8)	8 (3.5)
mAMR n (%)	2 (9.5)	37 (16.2)
**No molecular rejection n (%)**	18 (85.7)	158 (69.3)

Supplementary Table 7 │ Molecular rejection rates and differentiation – subgroups of biopsy indications: a) Demonstrates the Study Cohort (n=214), b) demonstrates the Positive Control Cohort (n=35). Categorical variables as numbers (n) and percentages (of the underlying group, %). The prefix «m» in front of every rejection phenotype stands for «molecular». *Abbreviations: TCMR, t cell-mediated rejection; AMR, antibody-mediated rejection.*

Supplementary Figures

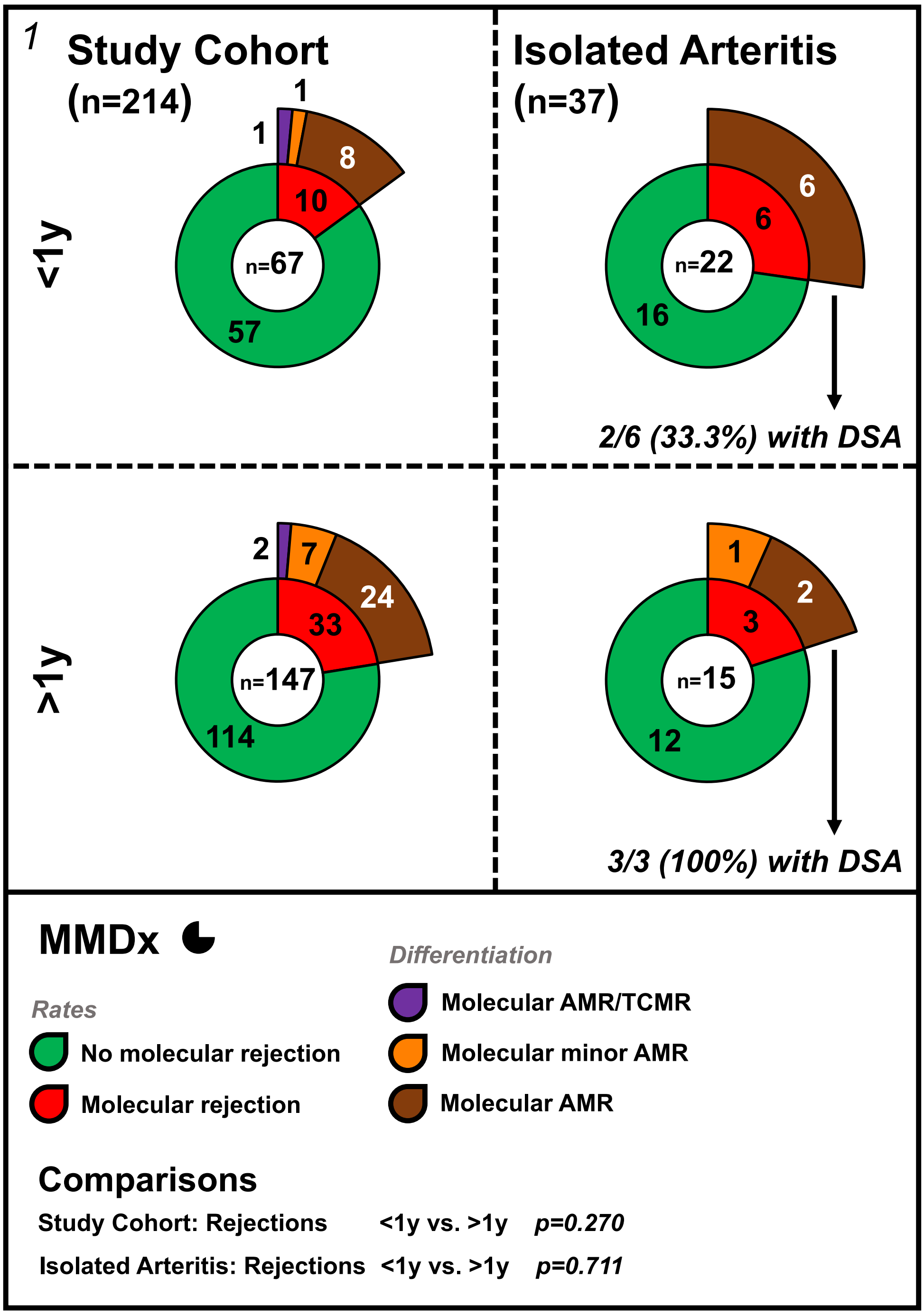

Supplementary Figure 1 │ Molecular rejection rates and differentiation – subgroups of time point: Molecular rejection rates and differentiation for the subgroups are separately presented based on biopsy before the first year of transplantation (<1y, above) or beyond the first year of transplantation (>1y, below). The Study Cohort and subgroup of Isolated Arteritis are visualized. Statistical comparisons are attached (Fisher's exact test).

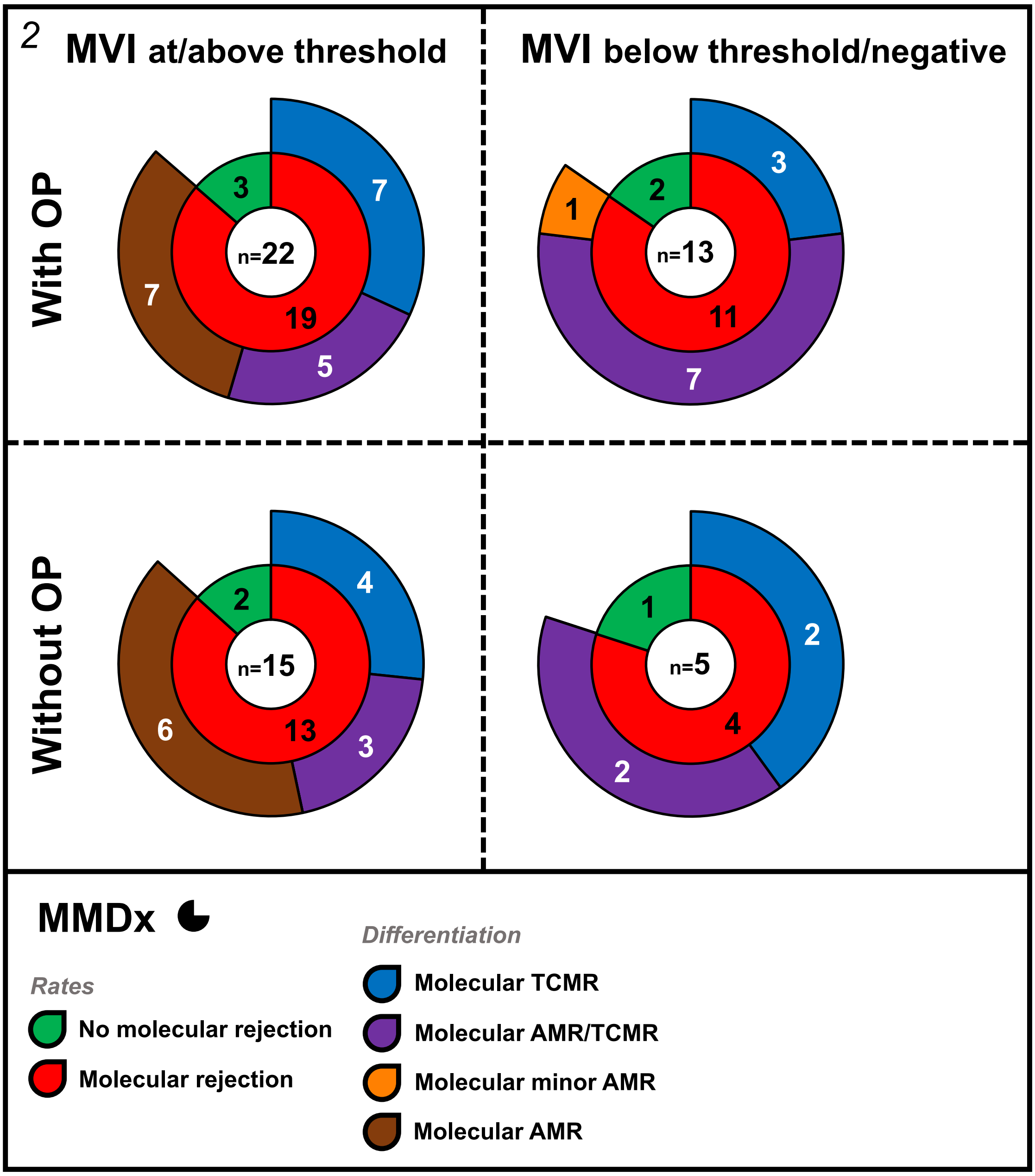

Supplementary Figure 2 │ Molecular rejection rates and differentiation – Positive Control Cohort with/without MVI: Molecular rejection rates and differentiation for the subgroups of the Positive Control Cohort (n=35) are separately presented based on MVI (microvascular inflammation) at/above threshold (left) or MVI below threshold/negative (right). Above are all cases are demonstrated (incl. OP, overlapping pathologies), below only the cases without OP are demonstrated. All full mAMR cases have MVI at/above threshold.

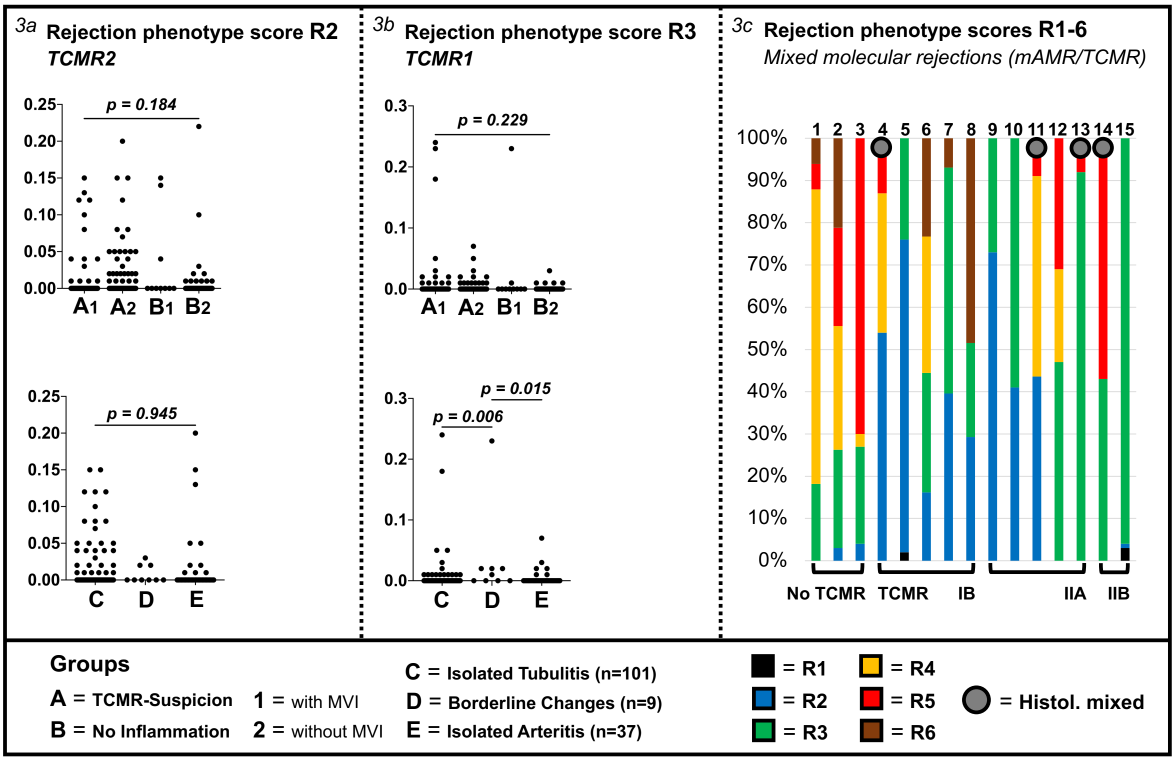

Supplementary Figure 3 │ Rejection phenotype scores of different subgroups: Figure 3a and 3b demonstrate the rejection phenotype scores (R2 and R3) for different subgroups as visible in the legend. Figure 3c reflect the rejection phenotype scores (R1-R6) for all 15 cases of mAMR/TCMR in the whole cohort (see Table 4 in the manuscript). R2 and R3 did not differentiate between MVI and no MVI (here meaning below threshold/negative). A small distinction of R3 is visible between group D (Borderline Changes) and the other groups. Regarding Figure 3c, different color patterns represent different percentages of R scores, however resulting in the same molecular phenotype of mAMR/TCMR.
